# MARVELD1 depletion leads to dysfunction of motor and cognition via regulating glia-dependent neuronal migration during brain development

**DOI:** 10.1038/s41419-018-1027-6

**Published:** 2018-09-24

**Authors:** Weizhe Liu, Fang Han, Shuai Qu, Yuanfei Yao, Jianxiang Zhao, Muhammad Luqman Akhtar, Yanpeng Ci, Hao Zhang, Hongfei Li, Yufang Zhao, Lei Yue, Yao Zhang, Changlin Wang, Yu Li

**Affiliations:** 1 School of Life Science and Technology, Harbin Institute of Technology, 150000 Harbin, China; 2 Department of Gastrointestinal Medical Oncology, Harbin Medical University Cancer Hospital, 150081 Harbin, China; 3 First college of clinical medicine, Nanjing university of Chinese medicine, 210000 Nanjing, China

## Abstract

The establishment of functional neuronal connectivity is dependent on the neuronal migration and the accurate positioning of neurons in the developing brain. Abnormal neuronal migration can trigger neuronal maturation defects and apoptosis. However, many genetic bases remain unclear in neuronal migration disorders during brain development. In this study, we reported that MARVELD1-defected mice displayed motor and cognitive dysfunction resulting from aberrant neuronal migration during brain development. The laminar organization of the cerebral cortex and cerebellum in MARVELD1 knockout (KO) mice is disrupted, indicating impaired radial neuronal migration. Furthermore, we used the cerebellum as a model to explore the radial neuronal migration processes, and the results demonstrated that the proper neuronal migration depended on MARVELD1 expression in glial cells of the developing brain. MARVELD1 suppressed the expression of ITGB1 and FAK Tyr397 phosphorylation in glia-dependent manner. The inhibition of the MARVELD1/ITGB1/FAK signalling pathway in MARVELD1 KO mice could reverse the defects in neuronal migration in vitro. Our findings revealed that MARVELD1 regulated neuronal migration by mediating the formation of glial fibres and ITGB1/FAK signalling pathway. The depletion of MARVELD1 during mouse brain development led to the abnormity of motor and cognition functions.

## Introduction

The emergence of mature neurons and the functional neuronal connectivity depends on neuronal migration in the developing brain^1–3^. Glia-guided radial migration ensures accurate positioning of major neurons as the most common migration pattern^4,5^. Newborn projection neurons in the cerebral cortex and granule cells in the cerebellum arrived at their target locations via this migrating pattern^1,2,6,7^. It is found that membrane-related signal events play crucial roles in the control of neuronal radial migration^5,8,9^. Some transduction paradigms of these signal molecules, such as interactions of cell adhesion molecules, have been illustrated as critical mechanisms underlying radial migration^1,8,9^. It is reported that ITGB1^1,10,11^, Astn 1/2^1,12^, N-cadherin^5,13^ and connexins^1,14^ can ensure the adhesion of the neuron to glial fibres during the process of neuronal radial migration. Moreover, the alternation of genes related to the radial migration could cause abnormal development and behavioural defects^2^^,^^15^. Thus, it is crucial to elucidate how neuronal radial migration is precisely regulated and which genes are involved in this process.

ITGB1 is a cell adhesion receptor, which is highly expressed in developmental brain of the mouse, and has been reported to regulate the rate of neuronal migration^10,11,16^. The cerebral cortical hemispheres and the cerebellum are reduced in size, and external foliation of the cerebellum is lacked in ITGB1 KO mice^17^. Moreover, glial fibres in ITGB1 KO mice are irregular and there is no anchorage at the cerebellar outer surface. Some research showed that conditional deletion of ITGB1 in astroglia might cause partial reactive gliosis^18^. Changing ECM elasticity with different ITGB1 content profoundly impacts on the ability of neuronal stem cells to undergo differentiation^19^. Meanwhile, ITGB1 binds to their ligands, which could induce alternation in the signals related to cellular migration and proliferation^10,16,20^.

MARVELD1 is a member of MARVEL domain-containing proteins, which is expressed in the cellular nucleus^21^. Previous studies showed that MARVELD1 expression is lower in tumour cells, and MARVELD1 regulates the balance of ITGB1 and ITGB4 expression in cancer cells^21–23^. It is known that MARVELD1 is barely expressed in normal adult human brain^21^. Ye Zhang also clarified that MARVELD1 was rarely expressed in the cerebral cortex of 7-day-old mice in both neurons and astrocytes^24^. But the expression and the function of MARVELD1 during brain development have not yet been fully identified. In this study, MARVELD1 KO mice and Nestin-cre/MARVELD1^fl/fl^ mice were utilized as the models to study the role of MARVELD1 during brain development. And it was found that MARVELD1 ablation led to mice behavioural and cognitive abnormity resulting from abnormal radial migration during brain development. Remarkably, the studies of GFAP-cre/MARVELD1^fl/fl^ mice show that MARVELD1 controlled precise positioning of neurons in the manner of glia-dependent by regulating the ITGB1/FAK signalling pathway.

## Results

### MARVELD1 KO mice displayed motor and cognitive function abnormalities

**Fig. 1:**
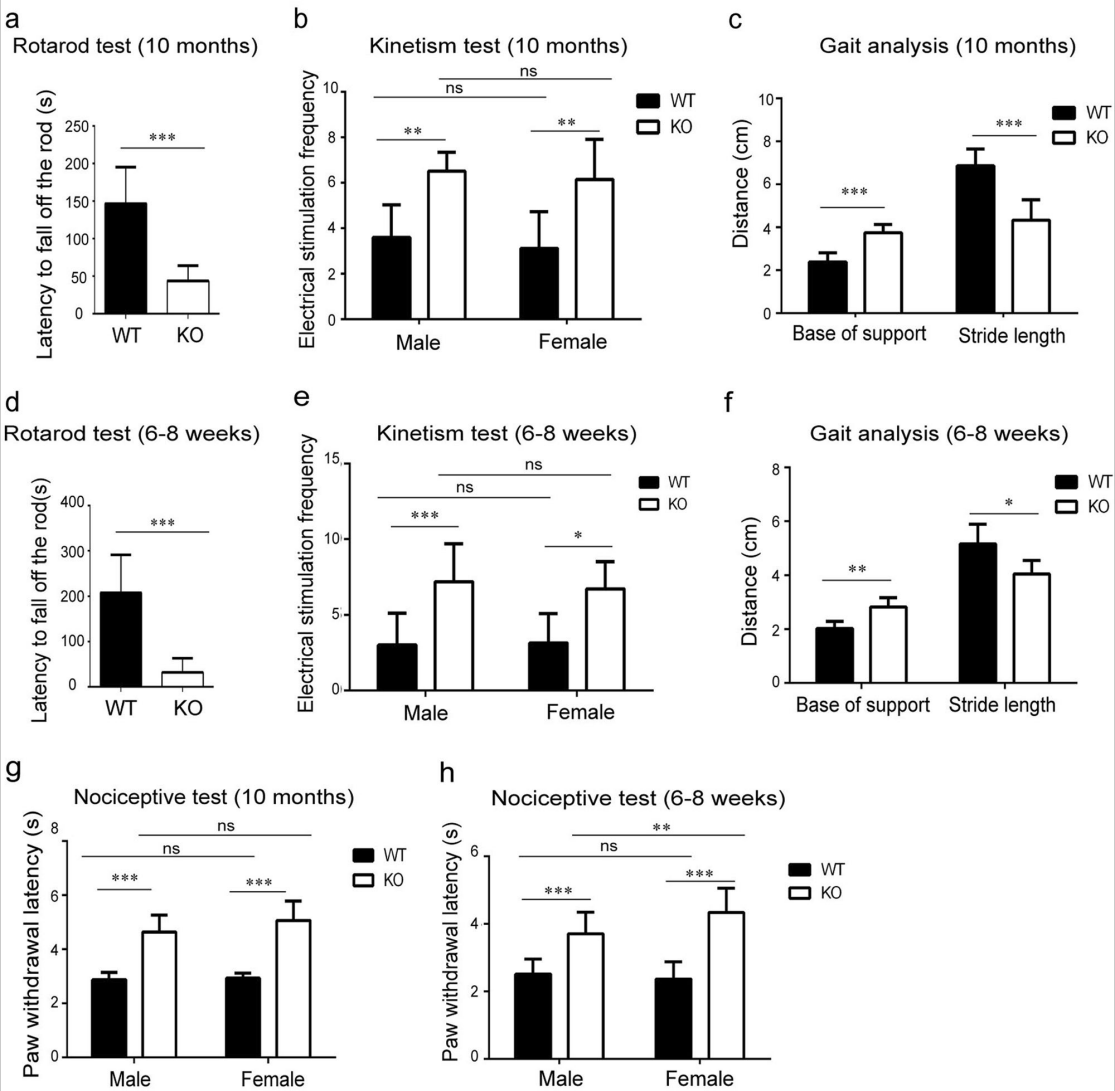
MARVELD1 KO mice displayed motor abnormalities. **a** Rotarod test was performed with 10-month-old mice. For WT mice *n* = 10, and for MARVELD1 KO mice *n* = 9. **b** Treadmill test: electrical stimulation frequency was analyzed using 10-month-old mice. For male mice: WT mice *n* = 10, and MARVELD1 KO mice *n* = 9; for female mice: WT mice *n* = 11, and MARVELD1 KO mice *n* = 9. One-way ANOVA was used in this study. **c** Gait experiment was tested using 10-month-old mice. Base of support and stride length were analyzed. For WT mice *n* = 12, and MARVELD1 KO mice *n* = 10. **d** Rotarod test was performed with 6–8-week-old mice. For WT and MARVELD1 KO mice, *n* = 13. **e** Treadmill test: electrical stimulation frequency was analyzed using 6–8-week-old mice. For male mice: WT and MARVELD1 KO mice *n* = 10, respectively; for female mice: WT and MARVELD1 KO mice *n* = 9, respectively. One-way ANOVA was used. **f** Gait test was tested using 6–8-week-old mice. Base of support and stride length were analyzed. For WT mice *n* = 11, and MARVELD1 KO mice *n* = 9. **g** The nociceptive response was assessed by the radiant heat paw withdrawal test using 10-month-old mice. For male mice: for WT mice *n* = 11 and for MARVELD1 KO mice *n* = 9; for female mice: for WT mice *n* = 10, and for MARVELD1 KO mice *n* = 9. One-way ANOVA was used. **h** The nociceptive response was assessed by the radiant heat paw withdrawal test using 6–8-week-old mice. For male mice: for WT mice *n* = 24 and MARVELD1 KO mice *n* = 27; for female mice: for WT mice *n* = 18 and MARVELD1 KO mice *n* = 23. One-way ANOVA was used. *p < 0.05; **p < 0.01; ***p < 0.001

To examine MARVELD1 function in the brain, a series of motor behavioural tests were performed on 10-month-old mice lacking MARVELD1 gene. For an initial measurement, rotarod test was carried out to estimate the balance and movement ability of the mice. MARVELD1 KO mice displayed a shorter latency to fall off the rod with 43.67 ± 6.82 s compared with 146.70 ± 15.35 s wild-type (WT) mice (*p* < 0.001) (Fig. 1a). In addition, the mouse treadmill assay exhibited a swing posture and a slower running speed in MARVELD1 KO mice, illustrating abnormal movement and balance ability (Fig. 1b). Then, in the gait experiment, it was observed that MARVELD1 KO mice with abnormal walking posture had a larger base of support and a closer stride length than the WT controls (Fig. 1c).

Moreover, to determine whether the abnormal motor phenotypes appeared in youthful MARVELD1 KO mice, the experiments were performed in 6–8-week-old mice. Consistent with results from the rotarod test, treadmill assay and gait analysis, it showed an impaired ability of balance, kinetism and movement in both 10-month-old and 6–8-week MARVELD1 KO mice (Fig. 1d–f). Meanwhile, the radiant paw withdrawal test showed that sensory behavioural dysfunctions were also found in both 10-month-old and 6–8-week-old MARVELD1 KO mice (Fig. 1g, h).

**Fig. 2:**
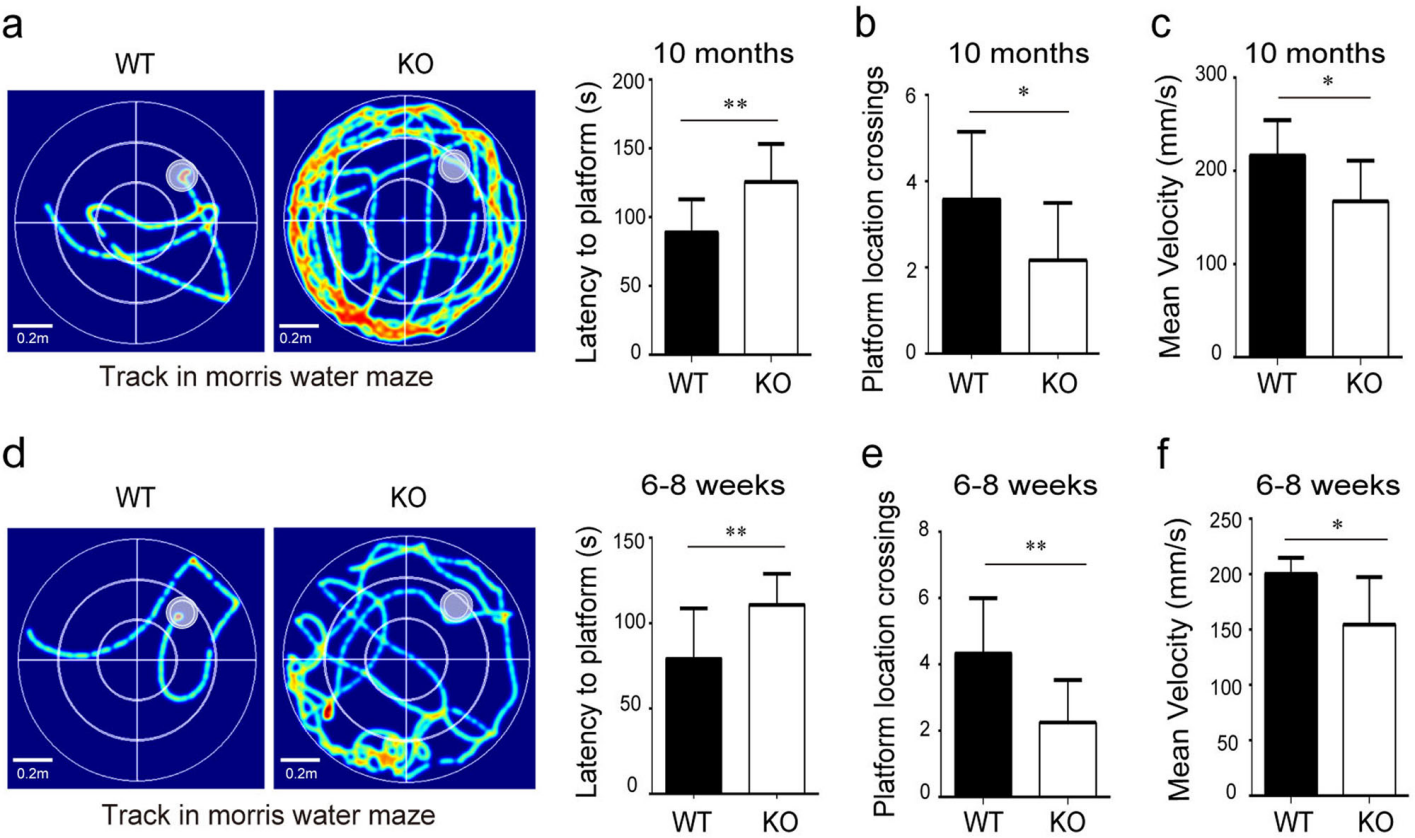
MARVELD1 KO mice displayed cognitive function abnormalities. **a** Morris water maze test was performed with 10-month-old mice. Training was conducted during 5 days with training two times per day and latency to the platform is analyzed. The platform was marked by a white pointed circle in the first quadrant. **b** A probe trial was assessed and the platform location crossing times were analyzed using 10-month-old mice. **c** The swimming velocity in Morris water maze test was analyzed using 10-month-old mice. The WT group and MARVELD1 KO group *n* = 11, respectively, in **a**, **b** and **c**. **d** Morris water maze test with 6–8-week-old mice and latency to the platform was analyzed. **e** A probe trial was assessed and the platform location crossing times were analyzed using 6–8-week-old mice. **f** The swimming velocity in Morris water maze test was analyzed using 6–8-week-old mice. For WT mice *n* = 13 and MARVELD1 KO mice *n* = 14 in **d**, **e** and **f**, respectively. *p < 0.05; **p < 0.01

Furthermore, the Morris water maze task was employed to evaluate abnormal spatial learning and memory abilities in 10-month-old MARVELD1 KO mice. The data showed that abilities of spatial learning and memory were significantly impaired in MARVELD1 KO mice compared with WT controls. The latency to arrive at the hidden platform was 56.04 ± 14.93 s in WT mice and 111.00 ± 6.04 s in MARVELD1 KO mice (*p* *<* 0.01) (Fig. 2a). Moreover, MARVELD1 KO mice displayed less times of crossing platform (*p* *<* 0.05) (Fig. 2b). The swimming velocity of MARVELD1 KO mice was slower than WT controls (Fig. 2c). The cognitive disorder was found in 6–8-week-old MARVELD1 KO mice as well (Fig. 2d–f). The results suggested that the motor and cognitive abnormity may occur in premature adults.

### The depletion of MARVELD1 led to neurodegeneration in mouse brain

**Fig. 3:**
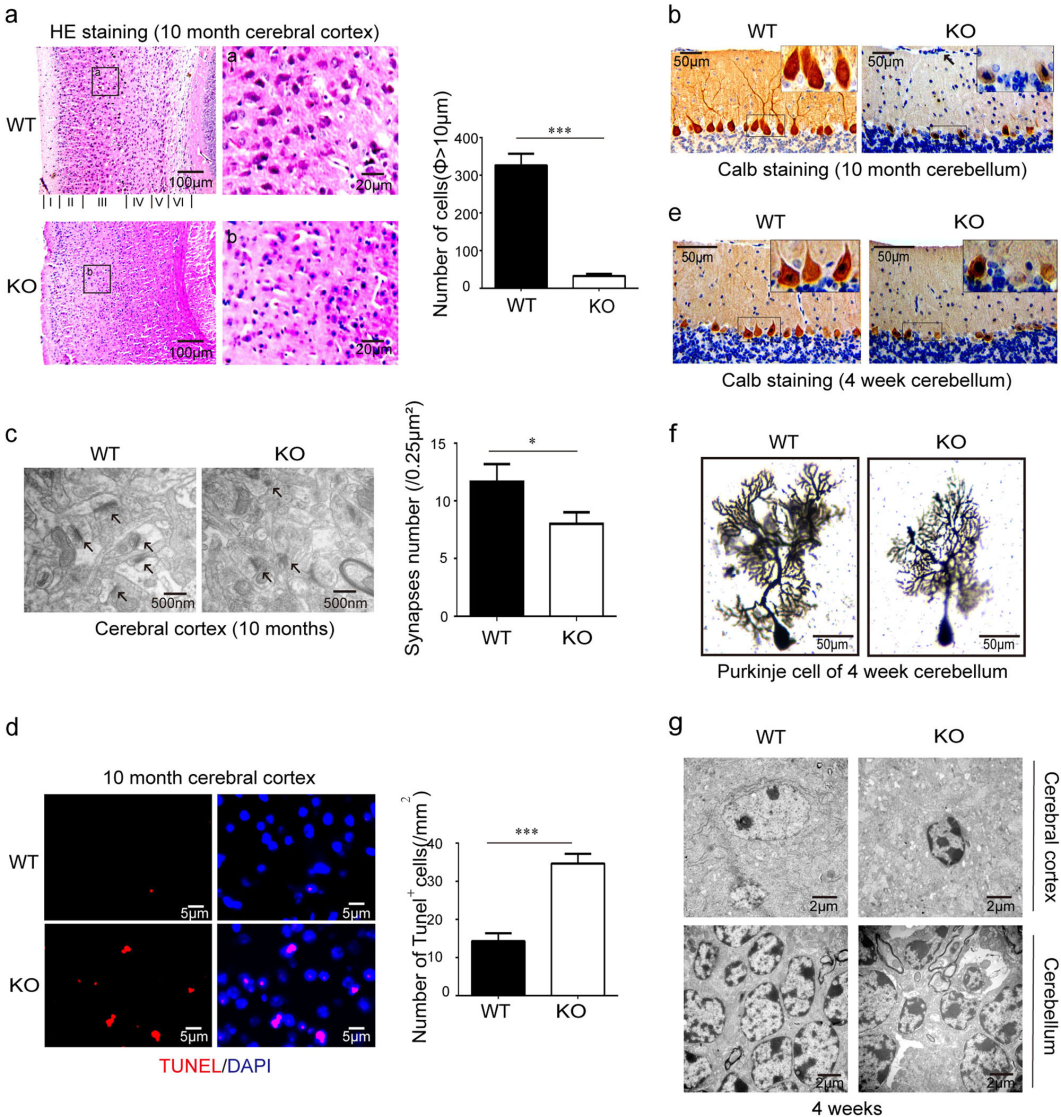
The depletion of MARVELD1 led to neurodegeneration in mouse brain. **a** The cerebral cortex HE staining of 10-month-old mice. Neurons with a diameter greater than 10  μm were counted (per mm^2^). **b** Immunohistochemistry staining was performed with Calb antibodies in 10-month-old mice cerebellum. **c** Transmission electron microscopy: neural synapses were observed in 10-month-old mice cerebral cortex. The arrowheads indicated neural synapses. **d** The number of TUNEL stained neurons/mm^2^ were counted in the cerebral cortex of 10-month-old mice. **e** Immunohistochemistry staining was performed with Calb antibodies in 4-week-old mice cerebella. **f** Golgi–Cox staining was performed to observe synapses of Purkinje cells in 4-week-old mice cerebellum. **g** Transmission electron microscopy: apoptotic neurons were observed in 4-week-old mice cerebral cortex and cerebellum. Above all investigations, *n* = 3 for each genotype. *p < 0.05; ***p < 0.001

Given the behavioural abnormality phenotype of old MARVELD1 KO mice, the tissue and cells of the cerebrum and the cerebellum were investigated. HE staining showed that there were numerous degenerating neurons in 10-month-old MARVELD1 KO mice (Fig. 3a). Statistical analysis indicated that there were fewer neurons in MARVELD1 KO mice with a diameter greater than 10 μm, especially in layer II, IV and V. In addition, the Purkinje cells in the cerebellum displayed notable atrophy in MARVELD1 KO mice (Fig. 3b), although no obvious morphological changes could be observed (Supplementary Fig. 1A). The results of transmission electron microscopy (TEM) further showed that the number of neural synapses was less and the structure was irregular in the cerebral cortex of MARVELD1 KO old mice (Fig. 3c). The TUNEL assay showed an increased number of apoptotic cerebral cortical cells in MARVELD1 KO mice (14.33 ± 1.20 in WT and 34.67 ± 1.45 in MARVELD1 KO mice per 1 mm^2^) (Fig. 3d).

Previous studies found that Purkinje cells completed their development process mainly in the 4th week of postnatal development^25–27^. However, immature Purkinje cells were observed in 4-week-old MARVELD1 KO mice (Fig. 3e). In the Golgi–Cox staining experiments, the results showed that the dendrites of Purkinje cells were notably sparse and shorter than WT controls (Fig. 3f). The dendrites in the MARVELD1 KO cerebral cortex were slender and disorderly compared with dendrites of WT mice as well (Supplementary Fig. 1B). Moreover, an early apoptosis in cerebral cortical neurons and cerebellar granule cells in 4-week-old MARVELD1 KO mice was also found (Fig. 3g). Also, there was an early apoptotic phenomenon in basket cells, a cell type that could form inhibitory synapses with Purkinje cells in the cerebellum^28^ (Supplementary Fig. 1C). To sum up, these abnormal phenotypes of MARVELD1 KO mice in the adult could trace back to brain developmental processes.

### Mice with MARVELD1 lack showed a laminar layer disorder in the cerebral cortex and cerebellum

**Fig. 4:**
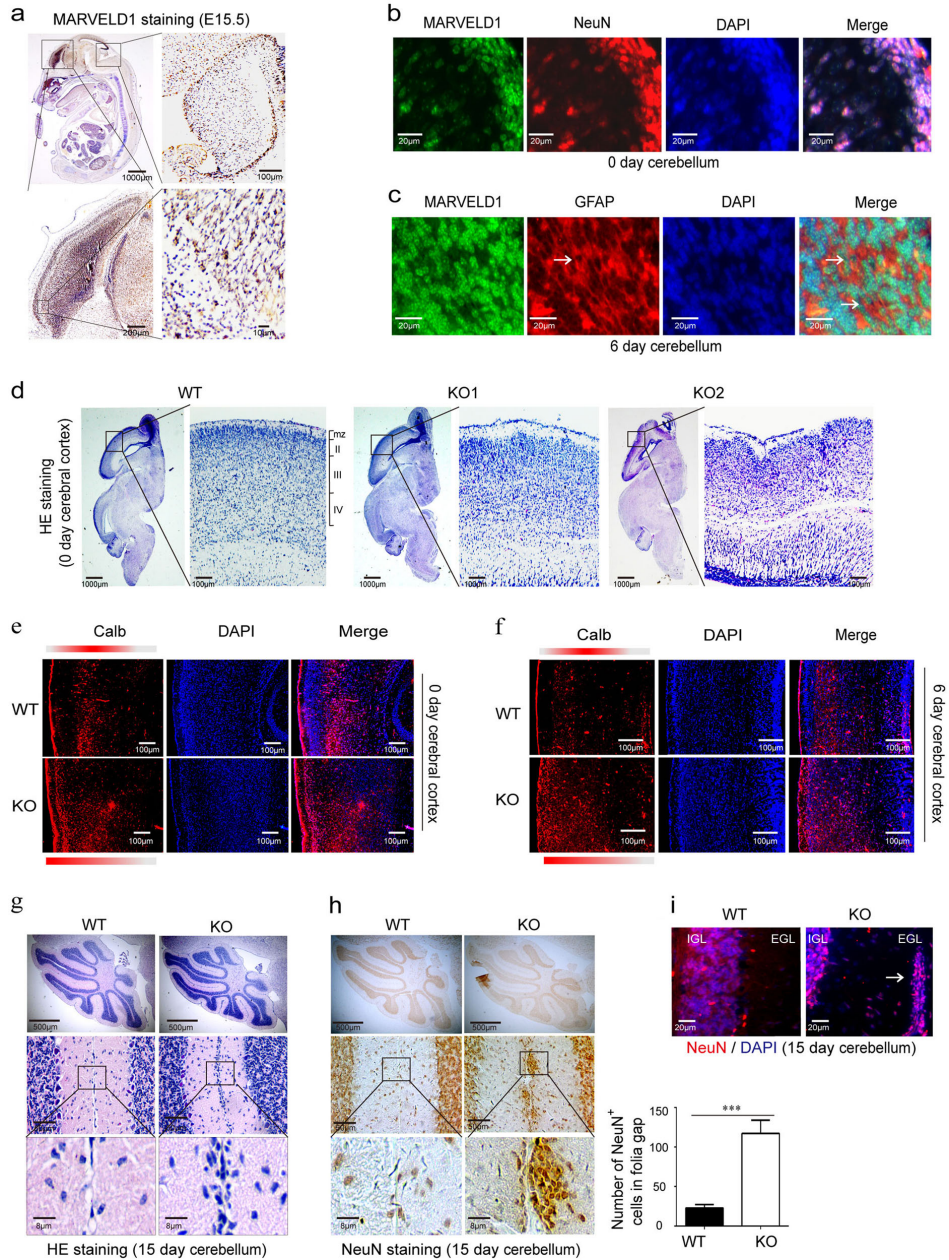
Mice with MARVELD1 lack showed a disorder of the laminar layers in the cerebral cortex and cerebellum. **a** Immunohistochemistry was observed for MARVELD1 protein from WT E15.5 mice. **b** Immunofluorescence was performed with antibodies of MARVELD1 (green) and a neuronal marker NeuN (red) in 0-day-old mice cerebellum. **c** Immunofluorescence was observed with antibodies of MARVELD1 (green) and glial cells maker GFAP (red) in 6-day-old mice cerebellum. **d** Sagittal sections through the cerebral cortex were analyzed by HE staining in 0-day-old mice. **e**–**f** Immunofluorescence of Calb (red) in 0-day-old and 6-day-old mice cerebral cortex. In control mice, Calb^+^ cells were present largely in laminaII/III and in MARVELD1 KO mice cerebral cortex they were distributed in superficial lamina or arranged in a disorderly manner. **g** Sagittal sections of 15-day-old mice cerebellum stained with HE. The whole cerebellum with low magnification shows the overall situation of abnormal cells location in the molecular layer. **h** Sagittal sections of 15-day-old mice cerebellum stained with immunohistochemistry of NeuN, a granule cell marker in the cerebellum. **i** Sagittal sections of 15-day-old mice cerebellum stained with immunofluorescence of DAPI and NeuN, a granule cell marker in the cerebellum. **d**–**i**: *n* = 3 for each genotype. ***p < 0.001

Based on the above results, MARVELD1 expression was examined in the embryo of WT mice. MARVELD1 was detected abundantly in embryonic brain, and its expression was obviously reduced after birth (Supplementary Fig. 2A, B). By in situ hybridization, MARVELD1 emerged in the brain of E 9.5 and E 10.5 mice (Supplementary Fig. 2C), and its expression was high in forebrain and midbrain in the E12.5~E15.5 mice (Supplementary Fig. 2D). Moreover, immunohistochemistry results also showed that MARVELD1 could be specifically detected in the brain (Fig. 4a). Meanwhile, immunofluorescence staining for a glial marker GFAP and a neuronal marker NeuN indicated that MARVELD1 was expressed in both glial cells and neurons in the cerebellum (Fig. 4b, c). These data suggested that MARVELD1 was highly expressed during the development of the cerebral cortex and the cerebellum.

The extrinsic feature and weight of the brain displayed no significant changes in the adult MARVELD1-deficient mice (Supplementary Fig. 2E). However, the cerebral cortex layers of MARVELD1 KO mice were disorganized and could not be defined accurately (Fig. 4d). In the MARVELD1 KO mice, the cerebral cortex neuroendocrine cells (marked by calbindin (Calb)) were arranged in a disorderly manner, indicating abnormal positioning of cerebral cortical neurons (Fig. 4e, f). HE staining experiment demonstrated an abundance of cellular accumulation throughout the molecular layer in 15-day-old MARVELD1 KO mice (Fig. 4g). Furthermore, dislocated cells could be granule cells, which were stained by NeuN (Fig. 4h, i). The neuronal dislocations in MARVELD1 KO mice represented an aberrant neuronal migration and maturation process.

Based on the consideration that the cerebellum is a good model to study the origin of migration deficits^4,29,30^, we focused on the cerebellum to explore the migrating process. In the cerebellum of 4-week-old Nestin-cre/MARVELD1^fl/fl^ mice, lots of cells were presented in the EGL as similar to MARVELD1 KO mice (Supplementary Fig. 3A). These abnormally located cells could be labelled with NeuN, which indicated that they were granule cells (Supplementary Fig. 3B, C). Meanwhile, glial scaffolds were severely disorganized and the fibre density was obviously reduced in Nestin-cre/MARVELD1^fl/fl^ mice. It was further found that the fibres were tiny and disarranged without connection or anchorage with the basement membrane (Supplementary Fig. 3D). Consistent with MARVELD1 KO mice, the shrinking cell bodies and a dramatic reduction of the apical dendritic arbour were observed in Purkinje cells (Supplementary Fig. 3E). Taken together, the results indicated the cerebellum abnormalities in both MARVELD1 KO and Nestin-cre/MARVELD1^fl/fl^ mice.

### MARVELD1 affected granule cell migration instead of proliferation

**Fig. 5:**
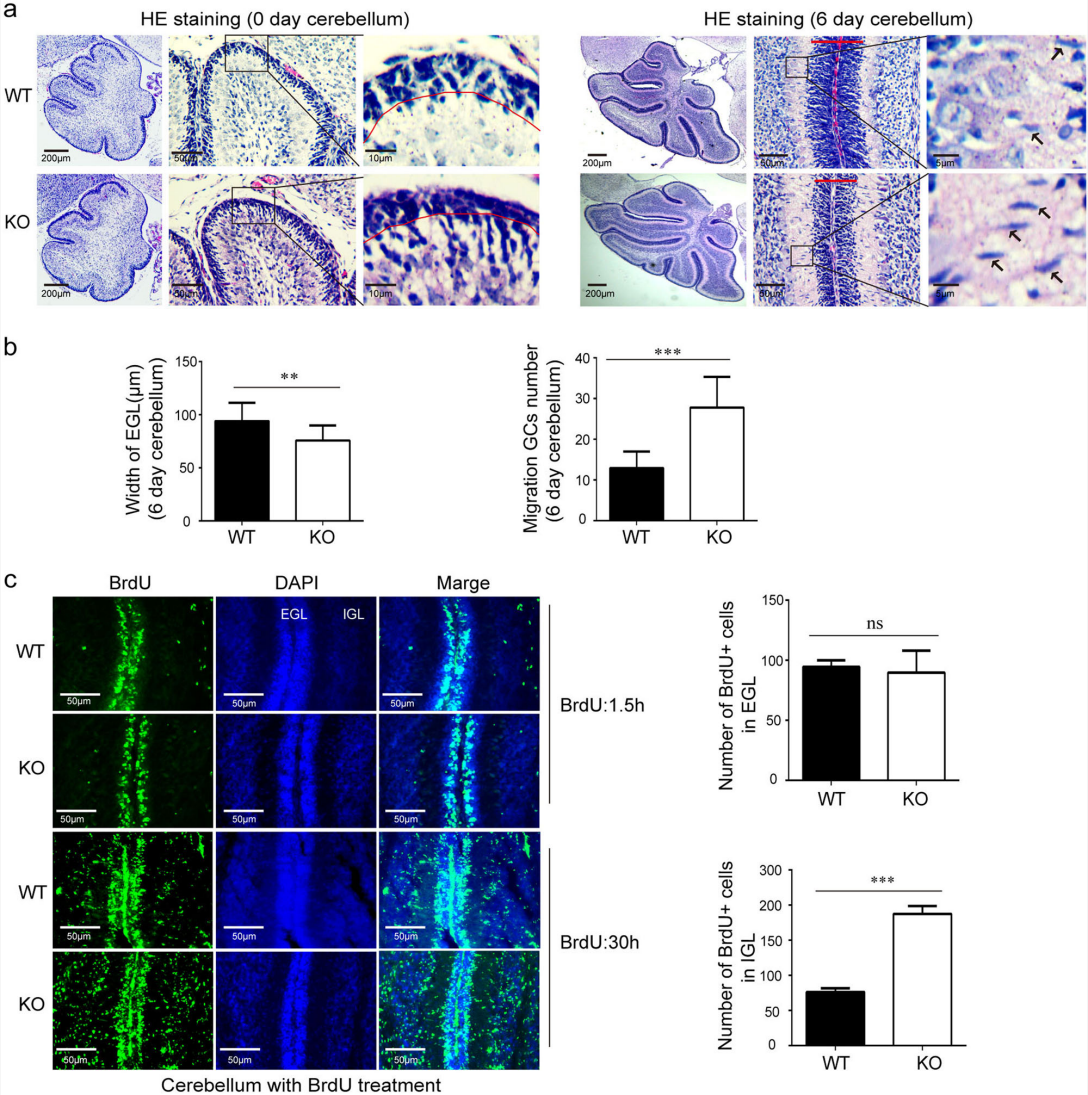
MARVELD1 affected granule cell migration but not proliferation. **a** Sagittal paraffin-embedded tissue sections of 0- and 6-day-old mice stained with HE. The whole cerebellum with low magnification showed the overall situation of abnormal cells. The arrowheads indicated migrating neurons. **b** In 6-day-old mice, the width of the EGL and the number of migrating granule cells were analyzed. **c** Granule cell proliferation and migration were evaluated after a short 1.5-h and a long 30-h chase following BrdU administration in 6-day-old mice in control and MARVELD1 KO animals. The relative cell number was counted. **a**–**c**: *n* = 3 for each genotype. **p < 0.01; ***p < 0.001

There was no clear boundary between the external granular layer and the Purkinje cell layer in the cerebellum of neonatal MARVELD1 KO mice (Fig. 5a). The width of the EGL also revealed a significant reduction in 6-day MARVELD1 KO mice (Fig. 5a, b). Consistent with an impaired inward radial migration in 0-day MARVELD1 KO mice, there were numerous migrating granule cells with characteristics of polarized and elongated shape in 6-day MARVELD1 KO mice.

Moreover, a notably increased number of BrdU^+^ cells in the internal granule cell layer (IGL) of MARVELD1 KO mice were found after a 30-h BrdU labelling, demonstrating the excessive cell migration in MARVELD1 KO mice. However, for proliferation, there were an approximately equal number of BrdU^+^ cells in the EGL of MARVELD1 KO mice compared with the WT controls after 1.5 h of BrdU labelling (Fig. 5c).

Sonic hedgehog (Shh) is the major mitogen that promotes granule cell proliferation during the development of the cerebellum^31–33^. RT-PCR analysis of cerebellar tissue lysates showed that Shh was slightly decreased in 7-day MARVELD1 KO mice. But the expression of the downstream genes, including Ptch1, N-myc and Gli1/2, was unaltered opposed to WT mice (Supplementary Fig. 4A−E). The data coincided with the equivalent number of BrdU^+^ neurons in the EGL of the MARVELD1 KO mice. Furthermore, genes related to neuronal migration were analyzed by RT-PCR. The neuronal adhesion molecule TAG1^34^ and neuronal protein astrotactin (Astn1)^12^ were unaltered (Supplementary Fig. 4F, G), but microtubule-associated protein DCX was increased in MARVELD1 KO mice (Supplementary Fig. 4H), which was highly expressed in migrating neurons^35^. The results verified that MARVELD1 ablation impaired granule cellular migration rather than proliferation.

### Abnormal migration of Bergmann glial cells restricted Purkinje cell maturation in MARVELD1 KO mice

Cerebellum developmental defects are followed by abnormality of Purkinje cell maturation^15,31^. In the cerebellum of 0-day-old MARVELD1 KO mice, the boundaries between the Purkinje cell layer and granule cell layer did not exist (Supplementary Fig. 5A). In the cerebellum of 6-day MARVELD1 KO mice, some Purkinje cells were disorganized in the Purkinje cell layer and aligned to the IGL (Supplementary Fig. 5B). Meanwhile, the width of the Purkinje cell layer was altered (Supplementary Fig. 5C). In 15-day-old MARVELD1 KO mice, abnormal Purkinje cells were also observed (Supplementary Fig. 5D).

Morphologically, glial cells in the cerebellum are mainly classified into three types: (i) bushy or velate protoplasmic astrocytes; (ii) smooth protoplasmic astrocytes and (iii) Bergmann glial cells^36,37^. Bergmann glial cells are highly polarized astrocytes extending massive radial fibres in the cerebellum^37–39^. The radial fibres of Bergmann glia serve as scaffolds for granule cell migration during postnatal development^4,40–42^. Furthermore, we observed the location of the Bergmann glial cells. The typical glial cell layer could be found in 0-day and 6-day WT mice. However, Bergmann glial cells were interspersed among the Purkinje cells and granule cells in MARVELD1 KO mice (Supplementary Fig. 5E−G). In addition, the results of immunohistochemical staining revealed that the glial fibres were sparse in 4-week-old MARVELD1 KO mice (Supplementary Fig. 5H). Together, the dislocated granule cells and stunted Purkinje cells were closely correlated with dislocation of Bergmann glial cells.

### MARVELD1 regulated accurate radial migration by affecting the formation of glial fibres

**Fig. 6:**
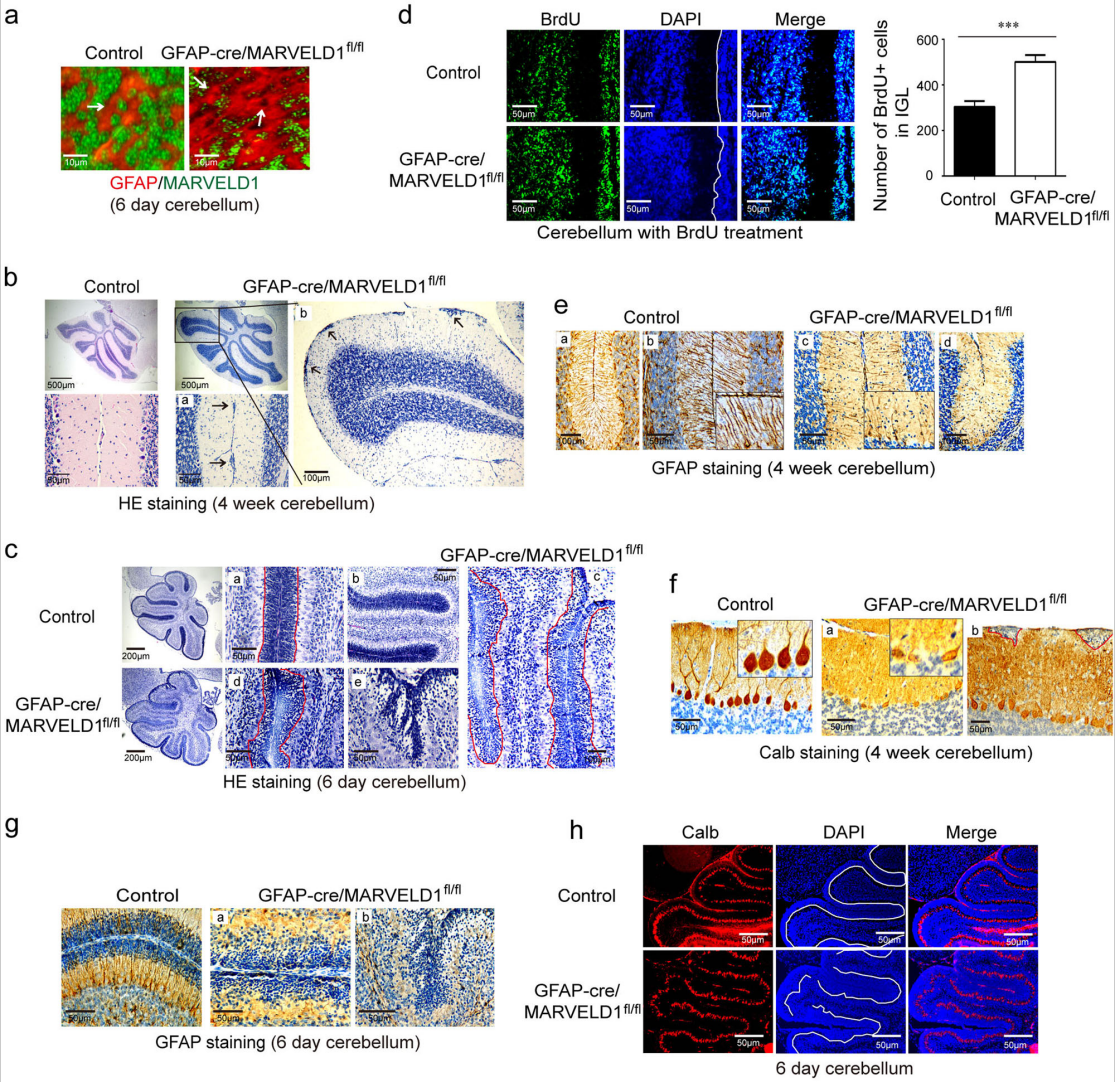
MARVELD1 regulated accurate radial migration by affecting the formation of glial fibres. **a** Immunofluorescence staining of MARVELD1 (green) and glial cells marker GFAP (red) in 6-day-old mice cerebellum. The arrowheads indicated glial cells. **b** HE staining of 4-week-old GFAP-cre/MARVELD1^fl/fl^ mice cerebellum sections. The whole cerebellum with low magnification showed the overall situation of abnormal cells location in the molecular layer. *n* = 3 for each genotype. **a** and **b** were different areas from GFAP-cre/MARVELD1^fl/fl^ cerebellum. **c** HE staining of 6-day-old control mice and GFAP-cre/MARVELD1^fl/fl^ mice cerebellum. The whole cerebellum with low magnification showed the overall situation of abnormal cells location in the molecular layer. *n* = 3 for each genotype. **a** and **b** were different areas from control cerebellum. **c**, **d** and **e** are different areas from GFAP-cre/MARVELD1^fl/fl^ cerebellum. **d** Granule cell migration was evaluated by a long 60-h chase following BrdU administration in 6-day-old control mice and GFAP-cre/MARVELD1^fl/fl^ mice. *n* = 3 for each genotype. **e** Immunohistochemistry staining with GFAP antibodies in 4-week-old control and GFAP-cre/MARVELD1^fl/fl^ cerebella. *n* = 3 for each genotype. **a** and **b** were different areas from control cerebellum. **c** and **d** were different areas from GFAP-cre/MARVELD1^fl/fl^ cerebellum. **f** Immunohistochemistry staining with Calb antibodies in control and GFAP-cre/MARVELD1^fl/fl^ cerebella in 4-week-old mice. *n* = 3 for each genotype. **a** and **b** are different areas from GFAP-cre/MARVELD1^fl/fl^ cerebellum. **g** Sagittal sections of 6-day-old mice cerebellum immunostained with anti-GFAP. GFAP-cre/MARVELD1^fl/fl^ mice had no obvious glial fibres. *n* = 3 for each genotype. **a** and **b** were different areas from GFAP-cre/MARVELD1^fl/fl^ cerebellum. **h** Immunofluorescence of Calb (red) in 6-day-old mice cerebellum. ***p < 0.001

To further determine whether the abnormal neuronal migration was interpreted by a neuronal intrinsic factor or by glial cells in MARVELD1 KO mice, GFAP-cre/MARVELD1^fl/fl^ mice were generated and there was no MARVELD1 expression in most of the glial cells in these mice (Fig. 6a). The results of HE staining showed that granule cells were accumulated in the molecular layer of the cerebellum and the result was the same with MARVELD1 KO mice and Nestin-cre/MARVELD1^fl/fl^ mice (Fig. 6b). In 6-day GFAP-cre/MARVELD1^fl/fl^ mice, granule cells were presented with an obvious disruption (Fig. 6c). The BrdU-labelling demonstrated an increased number of granule cells in the IGL in GFAP-cre/MARVELD1^fl/fl^ mice, which was similar to the migration defects in MARVELD1 KO mice (Fig. 6d).

In addition, the disorganized glial scaffold emerged in GFAP-cre/MARVELD1^fl/fl^ mice (Fig. 6e). It was found that maldevelopment of Purkinje cell apical dendritic arbours could not anchor into the basement membrane (Fig. 6f). On 6 day after birth, the GFAP-cre/MARVELD1^fl/fl^ mice had no strong glial fibres (Fig. 6g). Meanwhile, the Purkinje cell layer of GFAP-cre/MARVELD1^fl/fl^ mice was notably irregular (Fig. 6h). The results revealed that the dislocation of granule cells and the defects of Purkinje cells were derived from abnormity of Bergmann glial fibres instead of neurons.

The principal features of MARVELD1 KO mice and region-specific MARVELD1-deficient mice used throughout this study are summarized in Supplementary Table 1.

### MARVELD1/ITGB1/FAK signalling suppressed neuronal cell migration via glia-dependent manner

**Fig. 7:**
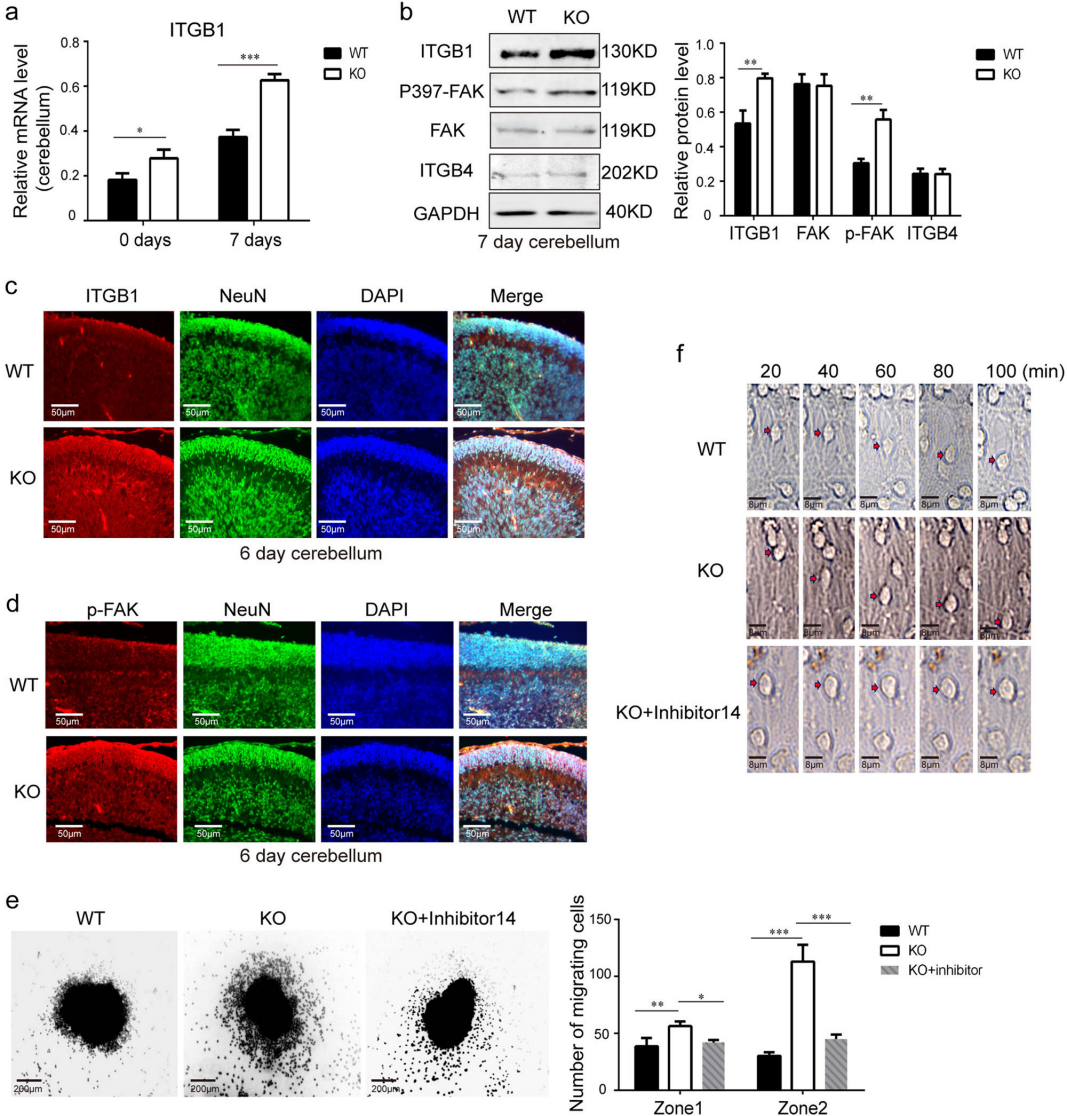
MARVELD1/ITGB1/FAK signalling suppressed neuronal cell migration via glia-dependent manner. **a** Quantitative analysis indicated increased levels of ITGB1 in 0- and 7-day-old mice. *n* = 3 for each genotype. One-way ANOVA was used in this study. **b** ITGB1 and FAK Tyr397 phosphorylation were detected by western blot in 7-day-old WT and MARVELD1 KO cerebellum whole lysates. Quantitative analysis indicates elevated ITGB1 and FAK Tyr397 phosphorylation levels in MARVELD1 KO mice. *n* = 3 for each genotype. **c** Immunofluorescence of ITGB1 (red) and NeuN (green) in 6-day-old mice cerebellum. **d** Immunofluorescence of p397-FAK (red) and NeuN (green) in 6-day-old mice cerebellum. **e** Neuron migration from 5-day-old mice microexplants of the cerebellum after 30 h was analyzed. DAPI staining revealed that there were more migrating granule cells in MARVELD1 KO mice and there was a reversion after adding an inhibiter (20 μM). The number of granule cells which had migrated to specified distances (zone1: 0–100  μm from the microexplants; zone2: 100  μm beyond) was analyzed. One-way ANOVA was used. **f** Time-lapse imaging series of migrating granule cells from 5-day-old explants cultured for 30 h before imaging. (Interval time between pictures is 20 min). *p < 0.05; **p < 0.01; ***p < 0.001

Our previous investigation found that MARVELD1 regulated the expression of ITGB1 and ITGB4 in cancer cells^22,23^. As shown in Fig. 7a and Supplementary Fig. 6A, ITGB1 mRNA notably increased in the cerebellar tissues of both 0- and 7-day MARVELD1 KO mice, whereas ITGB4 transcript levels remained unchanged. Furthermore, the results of western blot verified that ITGB1 protein level was increased in cerebellar tissues of MARVELD1 KO mice, but ITGB4 protein level was unchanged (Fig. 7b). This meant that MARVELD1 specially regulated ITGB1 rather than both ITGB1 and ITGB4 during brain development. Moreover, FAK, a downstream molecule of ITGB1^43^, was activated with MARVELD1 depletion. The elevated level of ITGB1 and FAK Tyr397 phosphorylation in neurons of MARVELD1 KO cerebellum was further identified by immunofluorescence staining (Fig. 7c, d).

Following exposure to the FAK inhibitor (20 μM), FAK Tyr397 phosphorylation decreased in primary neurons (Supplementary Fig. 6B). The cerebellum microexplants were performed, which included radial modes of neuronal migration^44–46^. The time-lapse results showed that the number and the distance of migrating cells after 30 h significantly increased in microexplants of MARVELD1 KO mice. After treatment with the FAK inhibitor, a reversion of both the migrating cell number and the migrating distance was observed in MARVELD1 KO neurons (Fig. 7e). As shown in Fig. 7f and the video, there were more migrating cells in MARVELD1 KO explants than WT controls. Meanwhile, the migrating distance of the MARVELD1 KO neuronal nucleus was longer. These migrating neuronal cells had less orientation compared with the WT migrating neuronal cells. The migrating distance and the number of cells were significantly reduced in FAK inhibitor-treated cells.

Using the immunofluorescence experiments, the increase of ITGB1 and FAK Tyr397 phosphorylation levels was further confirmed in the cerebellar neurons of GFAP-cre/MARVELD1^fl/fl^ mice, which suggested that the changes could be induced by MARVELD1 loss in glial cells (Supplementary Fig. 6C, D). To sum up, MARVELD1 regulated radial migration via ITGB1/FAK signalling pathway during brain development in a glia-dependent manner.

## Discussion

During ageing, some expressional changes were established reflecting regulatory patterns in the brain development. Recent studies in *C. elegans* had identified several developmental regulatory patterns that persisted into ageing and effectively limited the lifespan^47,48^. Interestingly, researchers proved that embryonic mutations of the *BRAF* gene in erythro-myeloid progenitors could cause the neurodegenerative disease after birth and induced the up-regulation of neurodegenerative markers in mouse^49,50^. Therefore, the link between developmental programmes and neurodegenerative disease has an objective existence. Despite the molecular bases about the relationship between brain development and neurodegeneration, there are still needs to be explored.

Initially, MARVELD1 was recognized for its role in cell proliferation and cell migration in tumour cells^21–23^. But the role of MARVELD1 in mouse brain development remains elusive. In this study, our results showed that MARVELD1 was essential for the radial neuronal migration during the brain development, and it played a significant role in motor and cognitive functions of the mouse. Cognitive abnormalities emerged in 6–8 weeks of MARVELD1 KO mice and neurons in both the cerebral cortex and the cerebellum of these mice showed apoptosis. Motor and cognitive abnormalities with MARVELD1 deletion could be continued from young to aged mice, so neurodegeneration and behavioural dysfunctions in aged MARVELD1 KO mice could be traced back to developmental stages. Thus, the study provided the linkage between brain development and neurodegeneration.

Integrins are a large family of cell adhesion receptors. ITGB1 is highly expressed during the brain development of mouse, and has a fundamental role in neuronal proliferation and radial migration^10,11,18,51,52^. Previous studies showed that MARVELD1 mediated the balance between ITGB1 and ITGB4 in cancer cells through down-regulating the expression of ITGB1 in mRNA processing and up-regulating ITGB4 by binding its promoter^21–23^. Another study showed that MARVELD1 specifically modulated ITGB4 during placental development^53^. In this research, it was found that MARVELD1 mediated neuronal migration via regulating ITGB1 rather than ITGB4 during brain development in mice. These results demonstrated that MARVELD1 regulated ITGB1 with tissue-specific pattern. The neuronal improper location was induced by overexpression of ITGB1 with MARVELD1 deletion, and led to neurodegeneration and behavioural abnormalities in MARVELD1 KO adults. These results support a linkage between the events during the brain development and the fate of a neuronal cell in ageing, and offer a new perspective to elucidate neurodegenerative diseases.

In the cerebral cortex, radial glia progenitor cells give rise to neurons and glia cells. Also they act as a scaffold for neuronal radial migration. So it is difficult to differentiate between potential glial and neuronal-specific defects. It is also difficult to determine either the disorganized laminar organization results from neurogenesis abnormalities or impaired migration. However, in the cerebellum, granule cells originated in the EGL and migrated inward to IGL along radial processes of Bergmann glia. Granule and glial cells originated from different precursors, which made it easier to establish the origin of migration deficits^4,29,30^. Based on these considerations, we focused on the cerebellum and used it as a model to research the migrating process.

Radially neuronal migration is the most common migration pattern in the neurons. This process not only participated in delivering the neurons to the appropriate place but was also involved in generating the laminar structure through successive neurogenesis and differentiation^54,55^. Radial migration in neurons is based upon glial fibres, so the defects in the glial cells could have a critical impact on accurate neuronal migration^56–58^. The results in GFAP-cre/MARVELD1^fl/fl^ mice demonstrated that MARVELD1 was specifically required in glial cells during cerebellar development. The data indicated that MARVELD1 had a non-cell autonomous role in granule cellular migration during the cerebellar development. MARVELD1 regulated radial migration via ITGB1/FAK signalling pathway in the brain development in a glia-dependent manner.

During cerebellar development, Purkinje cells secrete Shh to induce granule cell precursor proliferation^31–33^. In MARVELD1 KO mice, Purkinje cells have obvious defects in both the cell bodies and synapses, and their ability to secrete Shh was altered. In addition, we did not observe significant defects in granule cell precursor proliferation in MARVELD1 KO mice. The results suggested that MARVELD1 is not necessary for Shh signalling pathway in granule cell proliferation. Thus, it is proved that MARVELD1 precisely regulated the granule cellular migration, but not proliferation.

**Fig. 8:**
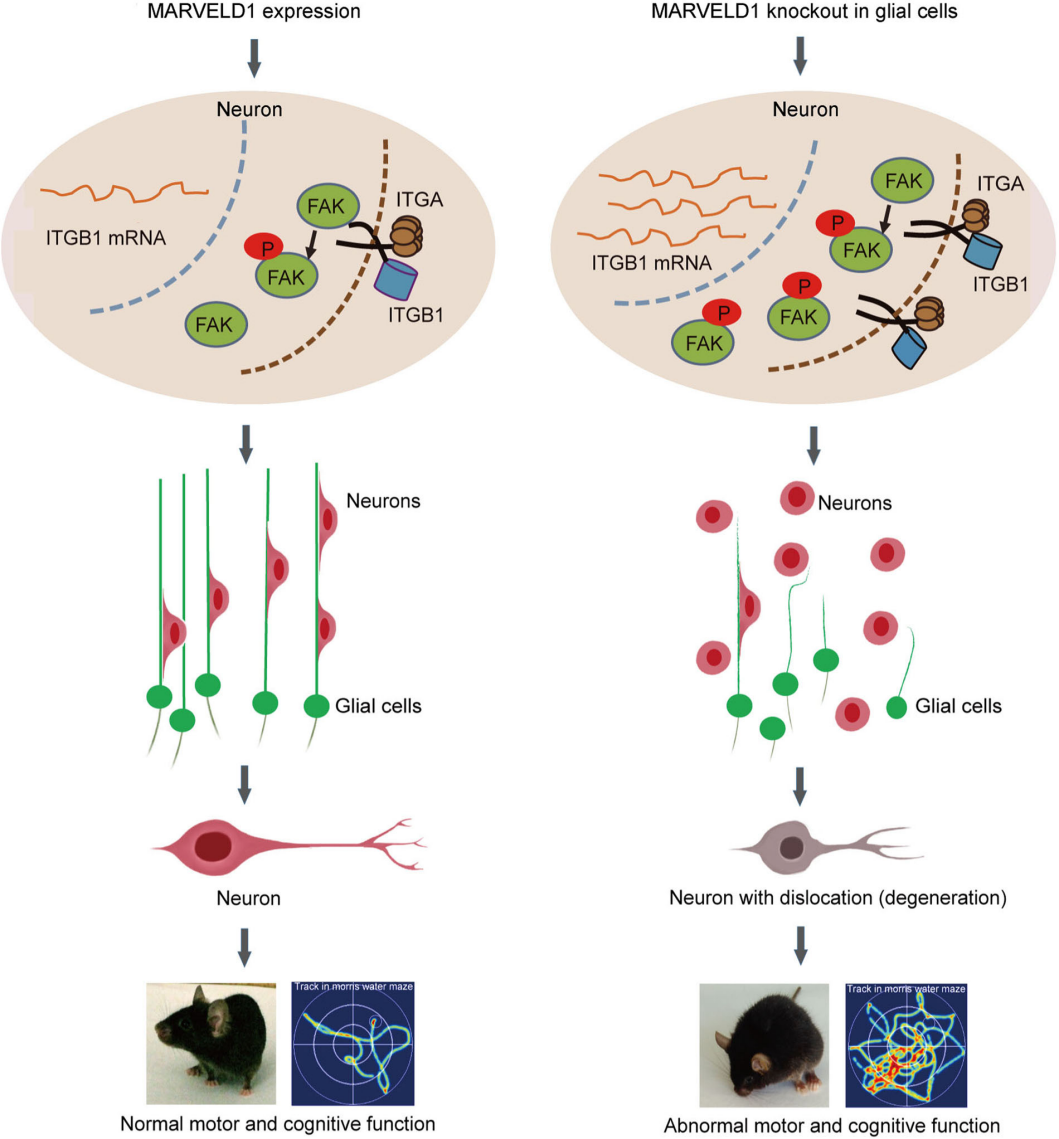
Schematic representation for MARVELD1-mediated neuronal migration. MARVELD1 deletion in glial cells induced the abnormality of glial fibres. Meanwhile, the expression of ITGB1 was increased in the pre-mRNA process in neurons, which further activated FAK through increasing its Tyr397 phosphorylation level. This regulation activated FAK-mediated downstream signalling that resulted in neuronal migration. The regulation process of MARVELD1 during brain development was in a glia cell-dependent manner. Furthermore, this process affected the neurodegeneration and behaviour in adult mice

Our study revealed that MARVELD1 deletion in glial cells induced the abnormality of glial fibres. Meanwhile, the expression of ITGB1 was increased and ITGB1/FAK signalling was activated in neurons. The changes in molecular level led to abnormal neuronal radial migration during the brain development in mice. The improper location and dysfunction of neurons in MARVELD1 KO mice might result in neurodegeneration and behavioural abnormalities in adults (Fig. 8).

## Materials and methods

### Animals

The whole body MARVELD1 KO mice and conditional KO mice (MARVELD1^fl/fl^) were generated by Biocytogen (China) in the C57BL/6J strain. For the whole-body MARVELD1 KO mice, the heterozygous mice were mated with WT C57BL/6J mice to cross out the cre allele, and the progeny was further backcrossed with C57BL/6J mice for at least eight generations. For the morphological experiments, all mice were littermate progeny from the heterozygous mice matings. For the conditional KO mice, the Nestin-cre^–/–^/MARVELD1^fl/fl^ or GFAP-cre^–/–^/MARVELD1^fl/fl^ littermate progeny animals served as controls. The transgenic mouse strain expressing the cre recombinase under the control of the glial fibrillary acidic protein promoter (GFAP-cre mice) was purchased from Shanghai Biomodel Organism Science & Technology Development Co. Ltd (FVB-Tg (GFAP-cre) 25Mes/J, Stock Number: 004600). In these mice, cre recombinase activity (as defined by expression of a floxed-STOP reporter gene) is targeted to most astrocytes throughout healthy brain and spinal cord tissues^59–61^. The transgenic mouse strain expressing the cre recombinase under the control of the Nestin promoter (Nestin-cre mice) was obtained from the Jackson Laboratory (B6.Cg-Tg (Nes-cre) 1Kln/J, Stock Number: 003771). Animals were housed in an animal room and room temperature was maintained at 24 ± 1 °C with a 12-h light and 12-h dark cycle. Food and water were available ad libitum. All animals were well cared and experiments were carried out in accordance with the principles and guidelines of the Ethics Committee of Harbin Institute of Technology.

### Behavioural experiment

Experiments for rotarod test, treadmill test and gait test were performed for WT and MARVELD1 KO mice (6–8-week and 10-month-old mice). The littermate progeny or non-littermate progeny were all used because of mice quantity requirements.

Rotarod test was performed as described in a previous investigation^62^ with some modification in this study. The animals were assessed in three habituation trials in 30-min intervals to stay on the rotating rod (ZB-200, Chengdu Taimeng Technology & Market Corporation, China) and a cut-off time of 300 s. The last time was utilized as the latency to fall off the rod. For 10-month-old mice: WT mice, *n* = 10 (including 6 males and 4 females); MARVELD1 KO mice, *n* = 9 (including 5 males and 4 females). For 6–8-week-old mice: WT mice, *n* = 13 (including 7 males and 6 females); MARVELD1 KO mice, *n* = 13 (including 6 males and 7 females).

In a treadmill test performed according to ref. ^63^ with additional modification, the speed of the transmission bands was 12 m/min. The mice with lower speed received electrical stimulation and the electrical stimulation frequency was analyzed. For 10-month-old mice: WT males, *n* = 10; MARVELD1 KO males, *n* = 9; WT females, *n* = 11; MARVELD1 KO females, *n* = 9. In a treadmill test in 6–8-week-old mice, for male mice: WT and KO mice *n* = 10, respectively; for female mice: WT and KO mice *n* = 9.

In gait test, mice were trained until they were able to go through the passageway without any pause. Training was conducted for 3 days twice a day. After finishing the training, the stride length and base of support of mice were analyzed^64^. For 10-month-old mice: WT mice, *n* = 12 (including 8 males and 4 females); MARVELD1 KO mice, *n* = 10 (including 5 males and 5 females). For 6–8-week-old mice: WT mice, *n* = 11 (including 5 males and 6 females); MARVELD1 KO mice, *n* = 9 (including 4 males and 5 females).

The nociceptive response was assessed by the radiant heat paw withdrawal test using a PL-200 radiant heat apparatus (Chengdu Taimeng Technology & Market Corporation, China), as described in a previous study^65^. The intensity of the radiant heat was defined as 60%, and the latency for paw withdrawal response against radiant heat stimulation was measured. In this experiment, for 10-month-old mice: WT males, *n* = 11; MARVELD1 KO males, *n* = 9; WT females, *n* = 10; MARVELD1 KO females, *n* = 9. For 6–8-week-old mice: WT males, *n* = 24; MARVELD1 KO males, *n* = 27; WT females, *n* = 18; MARVELD1 KO females, *n* = 23.

### Morris water maze

In total, 6–8-week and 10-month-old mice were used in this experiment. The Morris water maze test was performed as mentioned in Xie et al.^66^ with some modification. There were not <10 mice in each group. Briefly, a white, circular pool (1.2 m in diameter) was filled with water (22 °C) made opaque with nontoxicity, and a circular platform (10 cm in diameter) was submerged 1 cm beneath the surface of the water. The testing room was well lighted, and training was conducted for 5 days twice a day. The mice were placed randomly into the three starting quadrants except the quadrant on the platform. In each trial, the mice swam until they found the hidden platform or were guided to it, if they cannot find the hidden platform within 120 s. The mice remained on the platform for 10 s before being moved to the home cage. A probe trial was conducted on the 6th day. The hidden platform was removed, and mice were placed in the pool and allowed to swim for 120 s. For 10-month-old mice: WT mice, *n* = 11 (including 5 males and 6 females); MARVELD1 KO mice, *n* = 11 (including 6 males and 5 females). For 6–8-week-old mice: WT mice, *n* = 13 (including 8 males and 5 females); MARVELD1 KO mice, *n* = 14 (including 9 males and 5 females).

### Histology, immunohistochemistry and immunofluorescence

Tissues were fixed in 4% paraformaldehyde (PFA). Brains were either embedded in paraffin and sectioned to 4–5- μm sections or incubated with a high concentration of sucrose (30%) and rapidly frozen and sectioned at 8  μm. For immunohistochemistry and immunofluorescence, the following primary antibodies were used: anti-Calb (1:200, Sigma, no.sab4200543), anti-Blbp (1:100, Abcam, no.ab32423), anti-GFAP (1:200, Abcam, no.ab7260 or ab10062), anti-ITGB1 (1:100, Abcam, no.ab183666 or ab95623), anti-FAK (1:100, Abcam, no.ab40794), anti-p397-FAK (1:100, Abcam, no.ab81298), anti-BrdU (1:100, Sigma, no.b2531), anti-MARVELD1 (1:100, Abcam, no.ab91640 or no.ab169184) and anti-NeuN (1:200, Abcam, no.ab104224).

In immunohistochemistry study, paraffin-embedded sections were incubated in 3% H_2_O_2_ after microwave antigen retrieval. The sections were incubated with primary antibodies overnight at 4 °C. Then sections were incubated with biotinylated secondary antibodies for 1 h, followed by signal amplification and visualization with the avidin–biotin complex (ABC) system and DAB substrate (ZSGB Origene, Beijing, China). Zeiss microscope (Zeiss, Axio Zoom. V16) was used to detect the immunohistochemistry staining for sections in this study.

For immunofluorescence, the sections were incubated with primary antibodies overnight at 4 °C after microwave antigen retrieval and blocking in 3% bovine serum. Then sections were incubated with donkey anti-mouse IgG AlexaFluor-488 or donkey anti-mouse IgG AlexaFluor-568 (Invitrogen, Carlsbad, CA) for 1 h. All sections were counterstained with the nuclear marker DAPI (Burlingame, CA) for 15 min. A Zeiss fluorescence microscope (Zeiss, Axio Zoom. V16) was used to detect the fluorescence for sections in this study. The coverslips in Supplementary Fig. 7B were visualized by using Zeiss LSM 510 META confocal microscope.

### TUNEL assay

The TUNEL assay was performed as the protocol in TUNEL Assay kit (C10618, Invitrogen).

### Golgi–Cox staining

The Golgi staining was completed in accordance with the protocol provided by the neuron Golgi staining Kit (GenMed Scientifics Inc., USA). Briefly, the brain in 4- or 6-week-old mice was fixed and stained according to the manufacturer’s instructions. Tissues were sectioned at 30–80  μm and mounted on polylysine-coated slides. There were not less than 30 neurons analyzed for each genotype.

### Transmission electron microscopy

The cerebral cortex and the cerebellum were collected and cut into sections of 1–2-mm thickness. The tissues were fixed with glutaraldehyde for 12 h and osmic acid for 3 h at 4 °C. After washing, the specimens were dehydrated in 30–50–70–85–95% of ethanol, each for 20 min and twice in 100% ethanol for 30 min. The tissues were embedded in embedding agent including Epon812, DDSA, MNA and DMP-30. Then, the tissues were sectioned and stained. Neurons in the cerebral cortex and cerebellum were observed under the transmission electron microscope.

### BrdU pulse chase assays

Experiments were performed as described in a previous study^32^. BrdU was administered into neonatal mice intraperitoneally at a dosage of 200 mg per gram of body weight. Multiple BrdU pulses were performed at 12-h intervals. For analysis of cell proliferation, P6 mice were sacrificed 1.5 h after a single BrdU pulse. For the analysis of neuronal migration, animals were pulsed twice at P6 and sacrificed 30 h after the initial pulse. The sections were subjected to anti-BrdU fluorescence immunohistochemistry.

### Real-time PCR and Western blotting

Total RNA was extracted with the Trizol reagent (Invitrogen, Carlsbad, CA), and RNA was used for reverse transcription into complementary DNA (cDNA), using the PrimeScript TM RT-PCR Kit (Takara Biotechnology, Tokyo, Japan). Real-time PCR was performed on ViiATM7 Real-Time PCR System (Applied Biosystems, Foster City, CA), using the SYBR Premix Ex Taq II kit (Takara, Dalian, China). The primer sequences are listed in Supplementary Table S2.

For Western blotting, briefly, the cells were washed with ice-cold PBS and lysed with RIPA buffer (150 mM NaCl, 1% NP-40, 1% sodium deoxycholate, 0.1% SDS and 25 mM Tris-HCl, pH 7.6) containing protease inhibitors. The whole-cell lysates were electrophoresed in 12% SDS-PAGE and the protein was detected by an antibody. The following primary antibodies were used: anti-ITGB1 (1:1000, Abcam, no.ab183666), anti-ITGB4 (1:1000, Abcam, no.ab182120), anti-FAK (1:1000, Abcam, no.ab40794), anti-p397-FAK (1:800, Abcam, no.ab81298), anti-MARVELD1 (1:1000, Abcam, no.ab91640) and anti-GAPDH (1:2000, ZSGB, no.TA08).

### Microexplants culture, inhibitor and time-lapse experiments

Experiments were performed as described in the study^46^ and modified. Cerebellar microexplants were dissected from 5-day-old mice. Cerebella was aseptically dissected out and manually sliced into small tissue pieces. The sliced pieces were then manually transferred to pre-coated (100 g/ml poly-l-lysine) coverslips and placed in tissue culture dishes. The microexplants were cultured in medium containing 25 mM KCl and 10% calf serum in Basal Medium Eagle (BME, Gibco) plus penicillin–streptomycin–glutamine. The cultures were exposed to FAK inhibitor 14, a phosphorylation-specific inhibitor (20 μM, Sigma) for 24 h. In time-lapse experiments, neuronal cells around the microexplants were observed after being cultured for 30 h, and the interval time between pictures was 20 min.

### Statistical analysis

All quantitative variables were expressed as the mean ± SD. The statistical analyses were carried out using one-way ANOVA with Tukey’s multiple comparisons test or Student’s *t* test. One-way ANOVA was used in Fig. 1b, Fig. 1e, Fig. 1g, Fig. 1h, Fig. 7a, Fig. 7e, Supplementary Fig. 2A, Supplementary Fig. 4A-H, Supplementary Fig. 5C and Supplementary Fig. 6A. Student’s *t* test was used for others. All of the reported *p* values were two tailed, and *p* < 0.05 were considered statistically significant. The software Prism 7 (Graph Pad) was used for all statistical analyses.

## Electronic supplementary material

Supplemental Figure Legends

FigureS1

FigureS2

FigureS3

FigureS4

FigureS5

FigureS6

Table S1

Table S2

Supplemental video-WT

Supplemental video-KO

Supplemental video-KO+inhibitor

## References

[1] Evsyukova I, Plestant C, Anton ES (2013). Integrative mechanisms of oriented neuronal migration in the developing brain. Annu. Rev. Cell Dev. Biol..

[2] Riccardo Bocchi. (2017). Perturbed Wnt signaling leads to neuronal migration delay, altered interhemispheric connections and impaired social behavior. Nat. Commun..

[3] Sun D (2018). Neogenin, a regulator of adult hippocampal neurogenesis, prevents depressive-like behavior. Cell Death Dis..

[4] Jaarsma D (2014). A role for Bicaudal-D2 in radial cerebellar granule cell migration. Nat. Commun..

[5] Elias LAB, Wang DD, Kriegstein AR (2007). Gap junction adhesion is necessary for radial migration in the neocortex. Nature.

[6] Gang Chen. (2008). Semaphorin-3A guides radial migration of cortical neurons during development. Nat. Neurosci..

[7] Chedotal AShould (2010). I stay or should I go? Becoming a granule cell. Trends Neurosci..

[8] Cooper JA (2014). Molecules and mechanisms that regulate multipolar migration in the intermediate zone. Front. Cell Neurosci..

[9] Zhi Yang. (2017). ADAM10-initiated release of notch intracellular domain regulates microtubule stability and radial migration of cortical neurons. Cereb. Cortex.

[10] Belvindrah R, Graus-Porta D, Goebbels S, Nave KA, Müller U (2007). β1 integrins in radial glia but not in migrating neurons are essential for the formation of cell layers in the cerebral cortex. J. Neurosci..

[11] Anton ES, Kreidberg JA, Rakic P (1999). Distinct functions of α3 and αv integrin receptors in neuronal migration and laminar organization of the cerebral cortex. Neuron.

[12] Wilson PM, Fryer RH, Fang Y, Hatten ME (2010). Astn2, a novel member of the astrotactin gene family, regulates the trafficking of ASTN1 during glial-guided neuronal migration. J. Neurosci..

[13] Shikanai M, Nakajima K, Kawauchi T (2011). N-cadherin regulates radial glial fiber-dependent migration of cortical locomoting neurons. Commun. Integr. Biol..

[14] Elias LA, Turmaine M, Parnavelas JG, Kriegstein AR (2010). Connexin 43 mediates the tangential to radial migratory switch in ventrally derived cortical interneurons. J. Neurosci..

[15] Alvarez-Saavedra M (2014). Snf2h-mediated chromatin organization and histone H1 dynamics govern cerebellar morphogenesis and neural maturation. Nat. Commun..

[16] Huang Z (2006). Distinct roles of the β1-class integrins at the developing and the mature hippocampal excitatory synapse. J. Neurosci..

[17] Diana Graus (2001). β1-class integrins regulate the development of laminae and folia in the cerebral and cerebellar cortex. Neuron.

[18] Robel S (2009). Conditional deletion of beta1-integrin in astroglia causes partial reactive gliosis. Glia.

[19] Jing Du. (2011). Integrin activation and internalization on soft ECM as a mechanism of induction of stem cell differentiation by ECM elasticity. PNAS.

[20] Legate KR, Wickström SA, Fässler. R (2009). Genetic and cell biological analysis of integrin outside-in signaling. Genes Dev..

[21] Wang S (2009). Identification and characterization of MARVELD1, a novel nuclear protein that is down-regulated in multiple cancers and silenced by DNA methylation. Cancer Lett..

[22] Yao Y (2016). MARVELD1 modulates cell surface morphology and suppresses epithelial-mesenchymal transition in non-small cell lung cancer. Mol. Carcinog..

[23] Wang S (2013). MARVELD1 regulates integrin beta1-mediated cell adhesion and actin organization via inhibiting its pre-mRNA processing. Int. J. Biochem. Cell. Biol..

[24] Zhang. Ye (2014). An RNA-sequencing transcriptome and splicing database of glia, neurons, and vascular cells of the cerebral cortex. J. Neurosci..

[25] Li J (2010). Nna1 mediates Purkinje cell dendritic development via lysyl oxidase propeptide and NF-κB signaling. Neuron.

[26] Serra HG (2006). RORalpha-mediated Purkinje cell development determines disease severity in adult SCA1 mice. Cell.

[27] Goldowitz D, Hamre K (1998). The cells and molecules that make a cerebellum. Trends Neurosci..

[28] He Q (2015). Interneuron- and GABA (A) receptor-specific inhibitory synaptic plasticity in cerebellar Purkinje cells. Nat. Commun..

[29] Buffo A, Rossi F (2013). Origin, lineage and function of cerebellar glia. Prog. Neurobiol..

[30] Kriegstein A, Alvarez-Buylla A (2009). The glial nature of embryonic and adult neural stem cells. Annu. Rev. Neurosci..

[31] Dahmane N, Ruiz i. Altaba A (1999). Sonic hedgehog regulates the growth and patterning of the cerebellum. Development.

[32] Sanchez-Ortiz E (2014). NF1 regulation of RAS/ERK signaling is required for appropriate granule neuron progenitor expansion and migration in cerebellar development. Genes Dev..

[33] Cunningham D (2015). Analysis of hedgehog signaling in cerebellar granule cell precursors in a conditional Nsdhl allele demonstrates an essential role for cholesterol in postnatal CNS development. Hum. Mol. Genet..

[34] Kyriakopoulou K, de Diego I, Wassef M, Karagogeos D (2002). A combination of chain and neurophilic migration involving the adhesion molecule TAG-1 in the caudal medulla. Development.

[35] Gleeson JG, Lin PT, Flanagan LA, Walsh CA (1999). Doublecortin is a microtubule-associated protein and is expressed widely by migrating neurons. Neuron.

[36] Palay S, Chan-Palay V (1974). Cerebellar Cortex.

[37] Yamada K, Watanabe M (2002). Cytodifferentiation of Bergmann glia and its relationship with Purkinje cells. Anat. Sci. Int..

[38] Eiraku M (2005). DNER acts as a neuron-specific Notch ligand during Bergmann glial development. Nat. Neurosci..

[39] Yamada K (2000). Dynamic transformation of Bergmann glial fibers proceeds in correlation with dendritic outgrowth and synapse formation of cerebellar Purkinje cells. J. Comp. Neuro.

[40] Zheng C, Heintz N, Hatten ME (1996). CNS gene encoding astrotactin, which supports neuronal migration along glial fibers. Science.

[41] Rio C, Rieff HI, Qi P, Khurana TS, Corfas G (1997). Neuregulin and erbB receptors play a critical role in neuronal migration. Neuron.

[42] Driver AM, Shumrick C, Stottmann RW (2017). Ttc21b is required in bergmann glia for proper granule cell radial migration. J. Dev. Biol..

[43] Yang J (2016). Twist induces epithelial-mesenchymal transition and cell motility in breast cancer via ITGB1-FAK/ILK signaling axis and its associated downstream network. Int. J. Biochem. Cell Biol..

[44] Renaud J (2008). Plexin-A2 and its ligand, Sema6A, control nucleuscentrosome coupling in migrating granule cells. Nat. Neurosci..

[45] Kerjan G (2005). The transmembrane semaphorin Sema6A controls cerebellar granule cell migration. Nat. Neurosci..

[46] Kokubo M (2009). BDNF-mediated cerebellar granule cell development is impaired in mice null for CaMKK2 or CaMKIV. J. Neurosci..

[47] Somel M (2010). MicroRNA, mRNA, and protein expression link development and aging in human and macaque brain. Genome Res..

[48] de Magalhães JP, Church GM (2005). Genomes optimize reproduction: aging as a consequence of the developmental program. Physiology.

[49] Tarnawsky SP, Yoder MC (2017). From embryo mutation to adult degeneration. Nature.

[50] Idbaih A (2014). Dramatic response of a BRAF V600E-mutated primary CNS histiocytic sarcoma to vemurafenib. Neurology.

[51] Mills J (2006). Critical role of integrin-linked kinase in granule cell precursor proliferation and cerebellar development. J. Neurosci..

[52] Desgrosellier JS, Cheresh DA (2010). Integrins in cancer: biological implications and therapeutic opportunities. Nat. Rev. Cancer.

[53] Yue Chen (2014). The depletion of MARVELD1 leads to murine placenta accreta via integrin β4-dependent trophoblast cell invasion. J. Cell Physiol..

[54] Kay JN (2016). Kay. Radial migration: Retinal neurons hold on for the ride. J. Cell. Biol..

[55] Yohei Bamba. (2014). Differentiation, polarization, and migration of human induced pluripotent stem cell-derived neural progenitor cells co-cultured with a human glial cell line with radial glial-like characteristics. Biochem. Biophys. Res. Commun..

[56] Forster E (2002). Reelin, Disabled 1, and beta 1 integrins are required for the formation of the radial glial scaffold in the hippocampus. Proc. Natl Acad. Sci. USA.

[57] Belvindrah R (2006). Integrin-linked kinase regulates Bergmann glial differentiation during cerebellar development. Mol. Cell Neurosci..

[58] Ma S, Kw on HJ, Huang Z (2012). Ric-8a, a guanine nucleotide exchange factor for heterotrimeric G proteins, regulates bergmann glia-basement membrane adhesion during cerebellar foliation. J. Neurosci..

[59] Lang Zhuo (2001). hGFAP-cre transgenic mice for manipulation of glial and neuronal function in vivo. Genesis.

[60] Zhu L (2004). Non-invasive imaging of GFAP expression after neuronal damage in mice. Neurosci. Lett..

[61] Woosung Cho (2009). Dual transgenic reporter mice as a tool for monitoring expression of GFAP. J. Neurochem..

[62] Shiotsuki H (2010). A rotarod test for evaluation of motor skill learning. J. Neurosci. Methods.

[63] Sakurada S (2008). Possible involvement of dynorphin A release via mu1-opioid receptor on supraspinal antinociception of endomorphin-2. Peptides.

[64] Wang X (2017). Gait disorder as a predictor of spatial learning and memory impairment in aged mice. PeerJ.

[65] Wang CL (2017). Endomorphin-2 analogs with C-terminal esterification produce potent systemic antinociception with reduced tolerance and gastrointestinal side effects. Neuropharmacology.

[66] Xie H (2017). MiR-9 regulates the expression of BACE1 in dementia induced by chronic brain hypoperfusion in rats. Cell Physiol. Biochem..

