# Supplementary material for: MARVELD1 depletion leads to dysfunction of motor and cognition via regulating glia-dependent neuronal migration during brain development
Source: Cell Death Dis. 2018 Sep 24;9(10):999. doi: 10.1038/s41419-018-1027-6 (PMC6155261; doi:10.1038/s41419-018-1027-6)
Supplement: Supplementary file 1 — Supplemental Figure Legends [file 41419_2018_1027_MOESM1_ESM.docx]

**Supplemental Figure Legends**

**Figure S1 Aged MARVELD1 KO mice displayed neurodegeneration in brain. (A)** Sagittal sections of 10 month old mice cerebellum stained with HE. **(B)** Golgi-Cox staining was performed to observe synapse of neurons in 6 week old mice cerebral cortex. **(C)** Transmission electron microscopy: apoptotic neurons were observed in 4 week old cerebral cortex and cerebella. n=3 for each genotype.

**Figure S2 MARVELD1 was expressed during cerebral cortex and cerebellum development**. **(A)** Quantitative analysis indicated mRNA levels of MARVELD1 in whole brain of E12.5, E15.5 and 7 day old mice. n=3 for each time points. One-way-ANOVA was used here. **(B)** MARVELD1 expression was detected by western blot using whole brain lysates of E15.5, 7 day old mice, 2 week old mice and 4 week old mice. **(C)** In situ hybridization for MARVELD1 in WT E 9.5 and E 10.5 embryo. **(D)** In situ hybridization for MARVELD1 of E12.5-E15.5 embryonic sagittal sections. **(E)** The brain was dissected from 6 weeks WT and MARVELD1 KO mice. The weight for the whole brain and cerebellum of 6 week old mice was analyzed (n=11 for each genotype).

**Figure S3 The Nestin-cre/MARVELD1^fl/fl^ mice displayed the same disorganization of the cerebellum laminar layers as MARVELD1 KO mice**. **(A)** Sagittal sections stained with HE in 4 week old Nestin-cre/MARVELD1^fl/fl^ mice cerebellum. The whole cerebellum with low magnification showed the overall situation of abnormal cells location in the molecular layer. (a) and (b) were different area from MARVELD1 KO cerebellum. The arrowheads indicated the improper positioning cells. **(B)** Sagittal sections of 4 week old cerebellum stained with immunohistochemistry of NeuN, a granule cell maker in cerebellum. The arrowheads indicated the improper positioning cells. **(C)** Sagittal sections of 4 week old cerebellum stained with immunofluorescence of DAPI and NeuN. The arrowheads indicated the improper positioning cells in EGL of cerebellum. **(D)** Immunohistochemistry staining with GFAP antibodies in 4 week old control and Nestin-cre/MARVELD1^fl/fl^ cerebella. (a) and (b) were different area from MARVELD1 KO cerebellum. **(E)** Immunohistochemistry staining with Calb antibodies in 4 week old control and Nestin-cre/MARVELD1^fl/fl^ cerebella. (a) and (b) were different area from MARVELD1 KO cerebellum. **A, D** and **E**: n=3 for each genotype.

**Figure S4 MARVELD1 affected granule cell migration but not proliferation.** (**A-E**) RT-PCR analysis was performed for gene expression related to granule cell proliferation in 0 day and 7 day mice cerebellum. (**F-H**) RT-PCR analysis was performed for gene expression related to granule cell migration in 0 day and 7 day mice cerebellum. n=3 for each genotype. One-way-ANOVA was used here. * *p*< 0.05; ** *p*< 0.01; *** *p*< 0.001.

**Figure S5 Abnormal migration of Bergman glial cells restricted Purkinje cell maturation in MARVELD1** **KO mice. (A)** Immunohistochemistry staining was detected with Calb antibodies in 0 day mice cerebella. **(B)** Immunohistochemistry staining with Calb antibodies in 6 day mice cerebella. **(C)** The width of Purkinje cell layer was analyzed in both 0 and 6 day old mice. One-way-ANOVA was used here. **(D)** Immunohistochemistry staining with Calb antibodies in 15 day old mice cerebella. **(E)** Immunohistochemistry staining with Blbp antibodies in 0 day mice cerebella. **(F)** Immunofluorescence of Blbp (red) in 0 day mice cerebellum. **(G)** Immunofluorescence of Blbp (red) in 6 day old mice cerebellum. **(H)** Immunohistochemistry staining with GFAP antibodies in 4 week old WT and MARVELD1 KO cerebellum. **A-H**: n=3 for each genotype.

**Figure S6 MARVELD1/ITGB1/FAK signalling suppressed neuronal cell migration in a glia-dependent manner. (A)** Quantitative analysis indicated mRNA levels of ITGB4 in 0 day and 7 day mice brain. n=3 for each genotype. One-way-ANOVA was used here. **(B)** Immunofluorescence of FAK Tyr397 phosphorylation in 5 day microexplants after 30 h. FAK Tyr397 phosphorylation was suppressed after adding inhibiter (20 μM). **(C)** Immunofluorescence of ITGB1 (red) and NeuN (green) in GFAP-cre/MARVELD1^fl/fl^ cerebella of 6 day mice. **(D)** Immunofluorescence of p397-FAK (red) and NeuN (green) in GFAP-cre/MARVELD1^fl/fl^ cerebella of 6 day mice.

**Table S1 Principal features of specific MARVELD1 deficient mice used in this study**

**Table S2 Sequences of oligonucleotides used in Real Time PCR**
