# Supplementary figures and images for: MARVELD1 depletion leads to dysfunction of motor and cognition via regulating glia-dependent neuronal migration during brain development
Source: Cell Death Dis. 2018 Sep 24;9(10):999. doi: 10.1038/s41419-018-1027-6 (PMC6155261; doi:10.1038/s41419-018-1027-6)

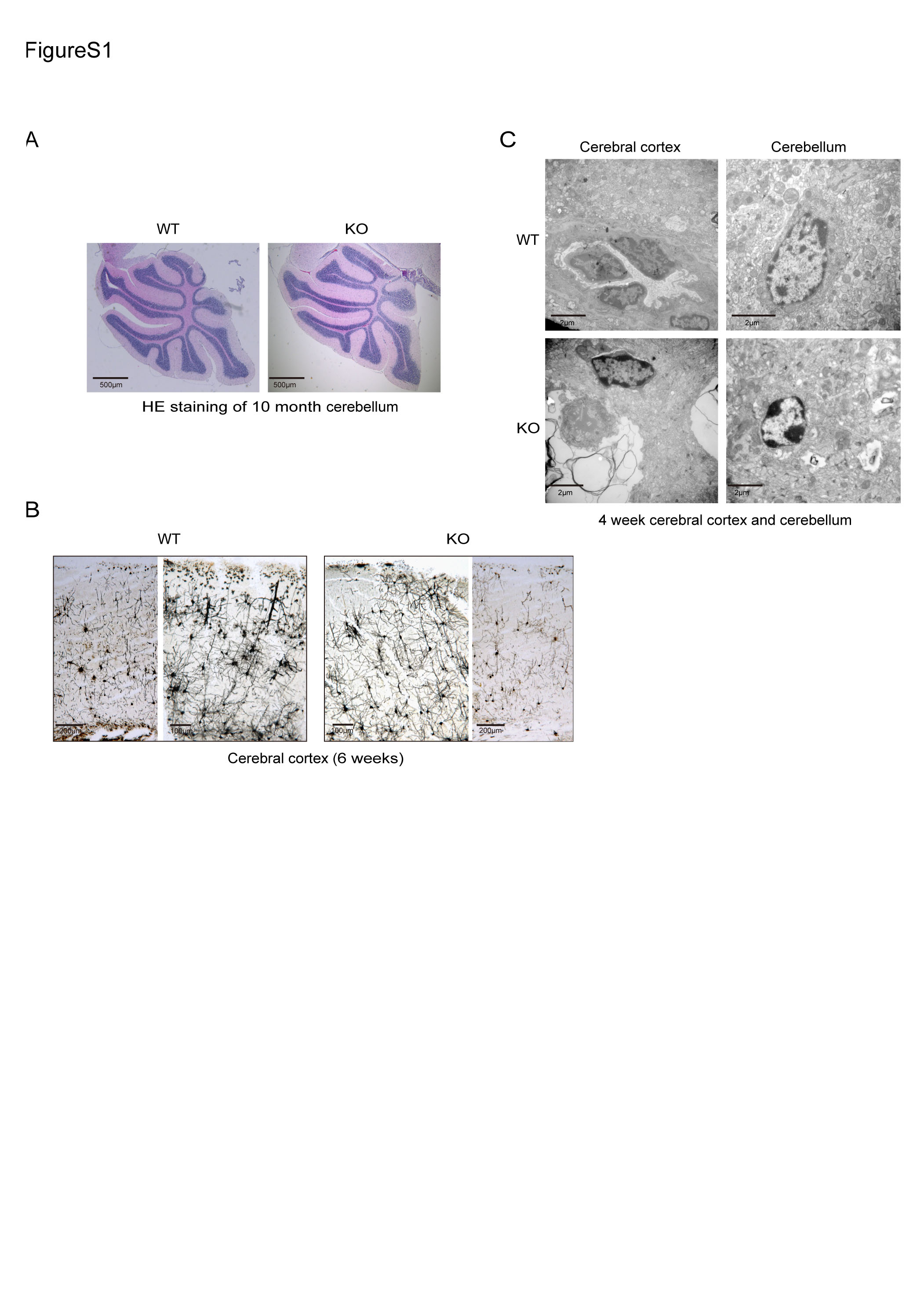

Supplement: Supplementary file 2 — FigureS1 [file 41419_2018_1027_MOESM2_ESM.jpg]

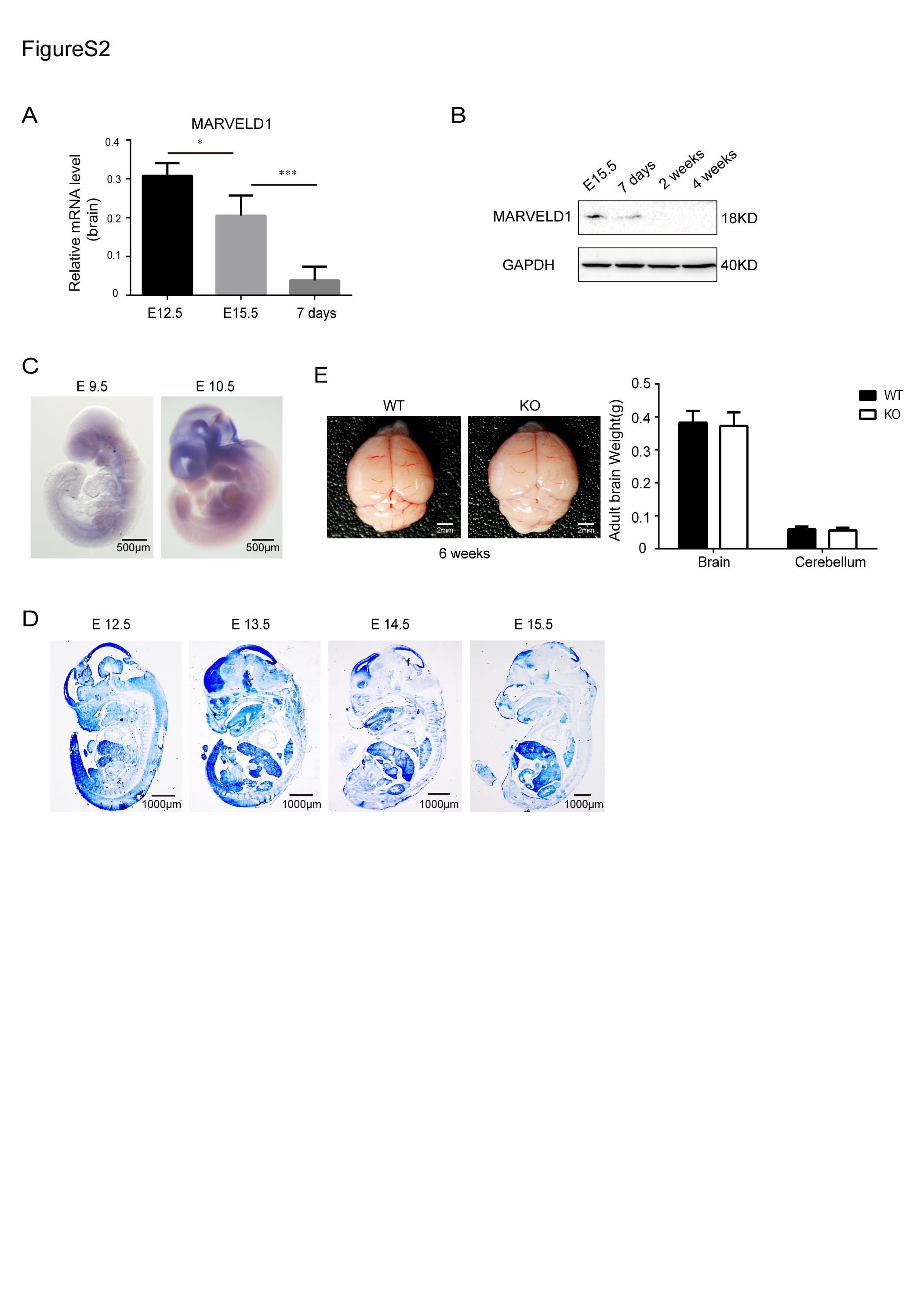

Supplement: Supplementary file 3 — FigureS2 [file 41419_2018_1027_MOESM3_ESM.jpg]

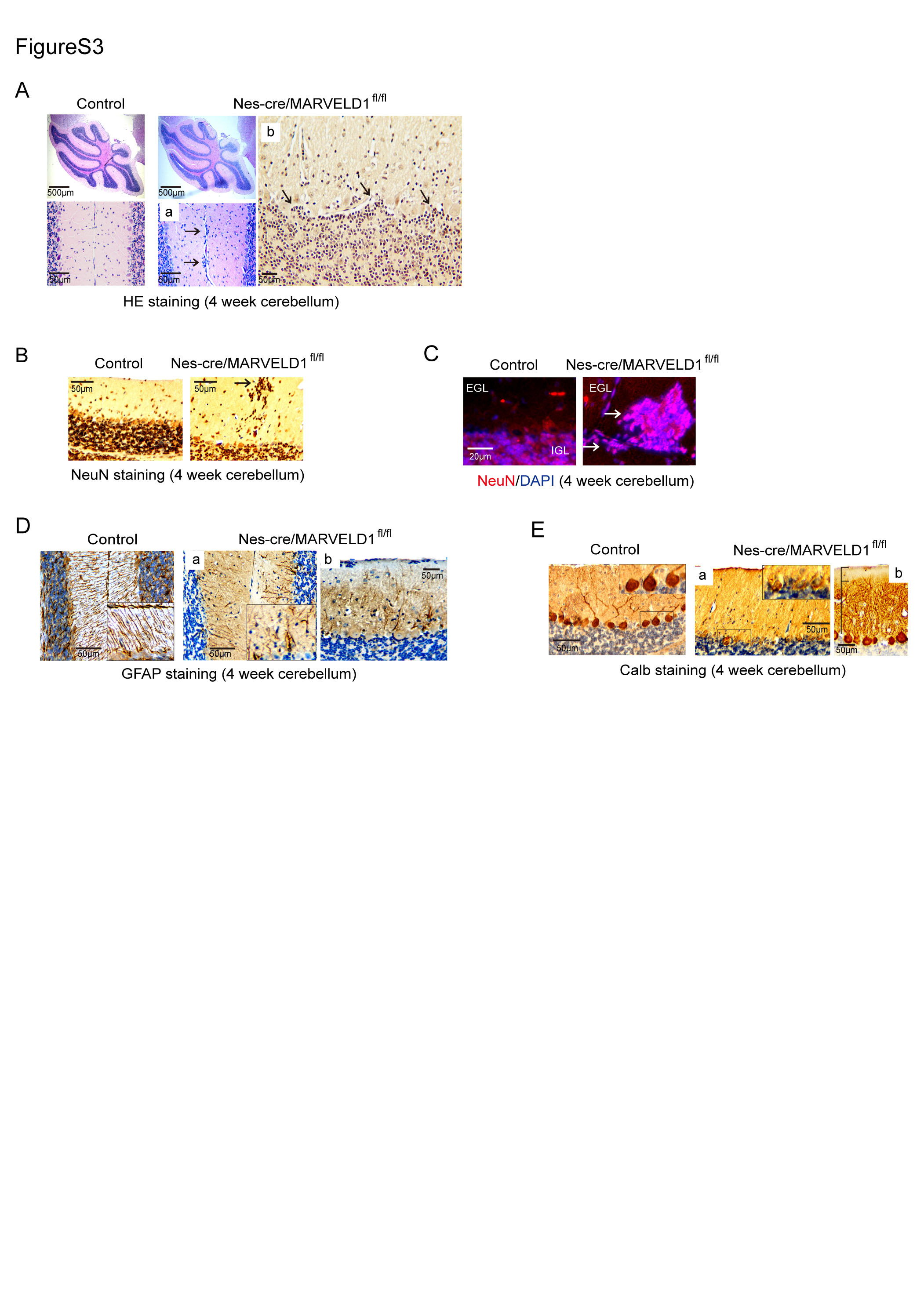

Supplement: Supplementary file 4 — FigureS3 [file 41419_2018_1027_MOESM4_ESM.jpg]

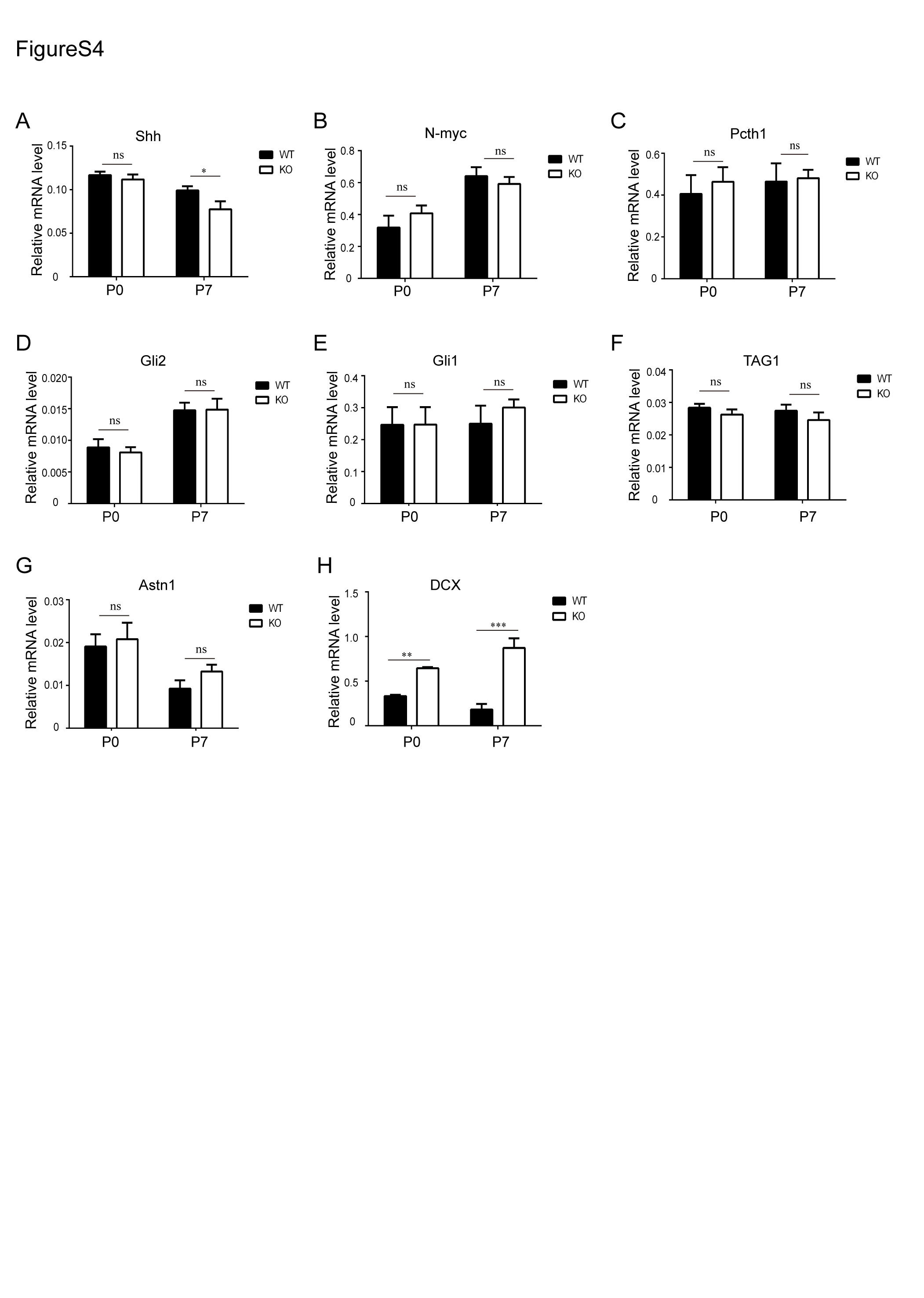

Supplement: Supplementary file 5 — FigureS4 [file 41419_2018_1027_MOESM5_ESM.jpg]

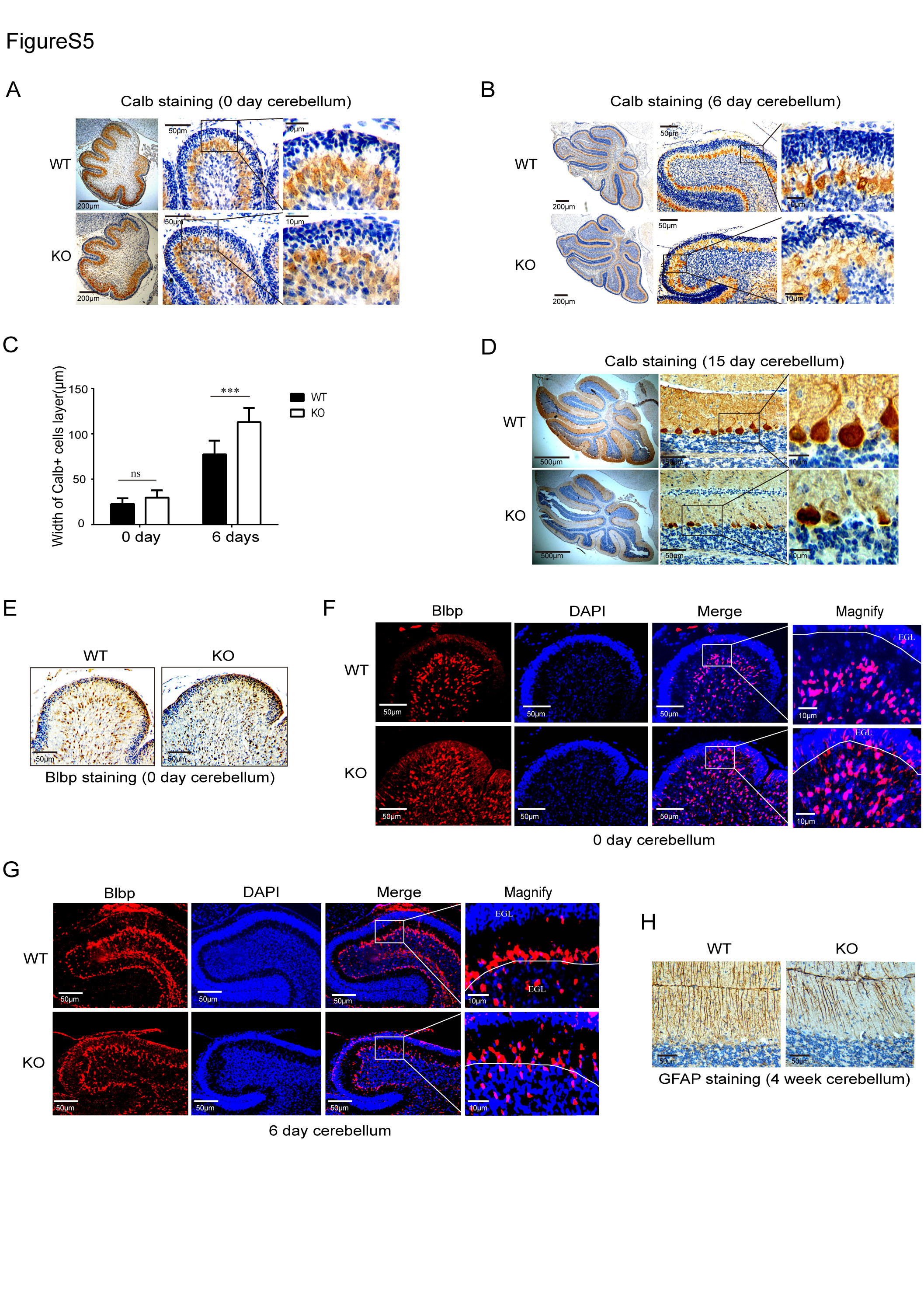

Supplement: Supplementary file 6 — FigureS5 [file 41419_2018_1027_MOESM6_ESM.jpg]

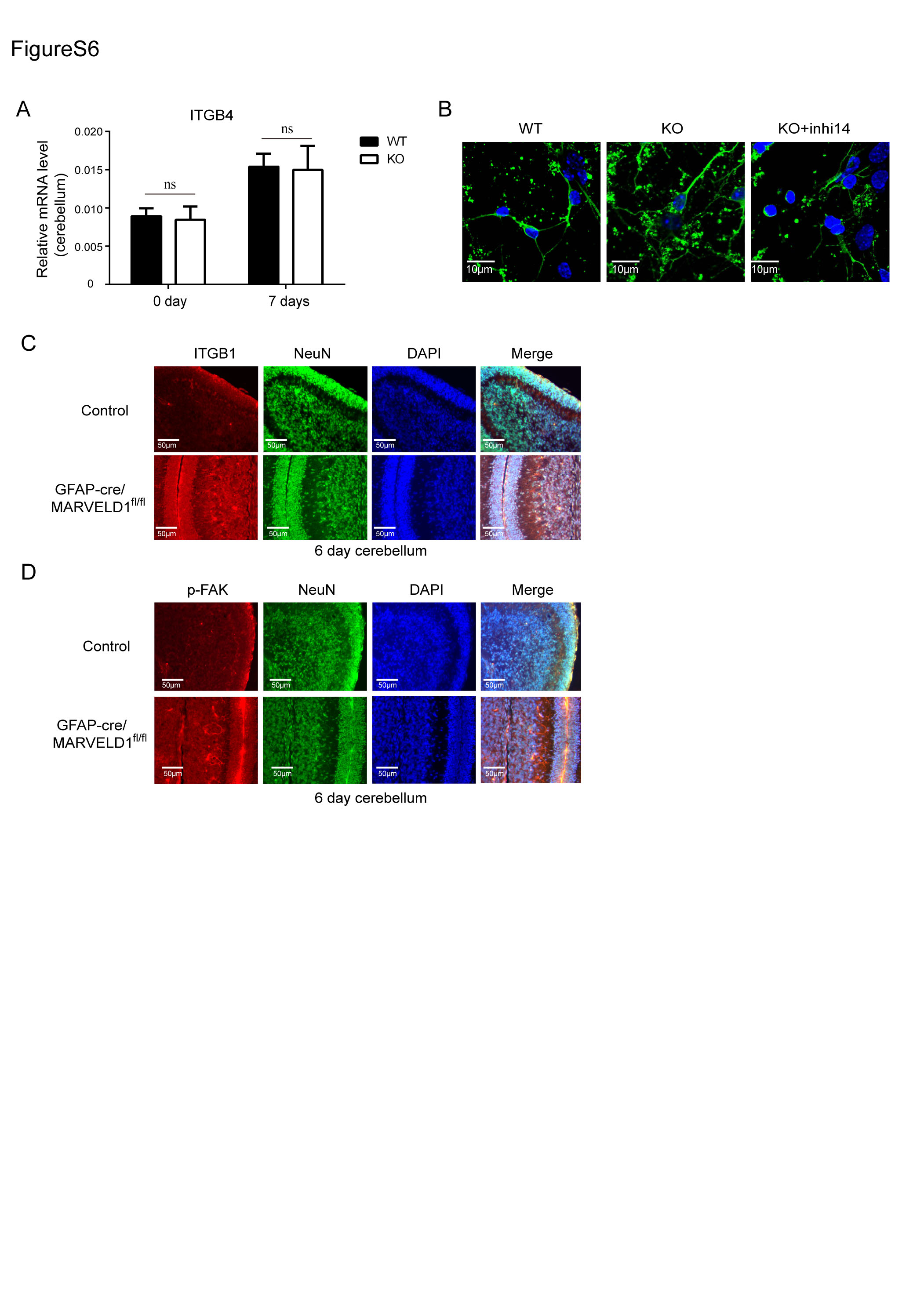

Supplement: Supplementary file 7 — FigureS6 [file 41419_2018_1027_MOESM7_ESM.jpg]

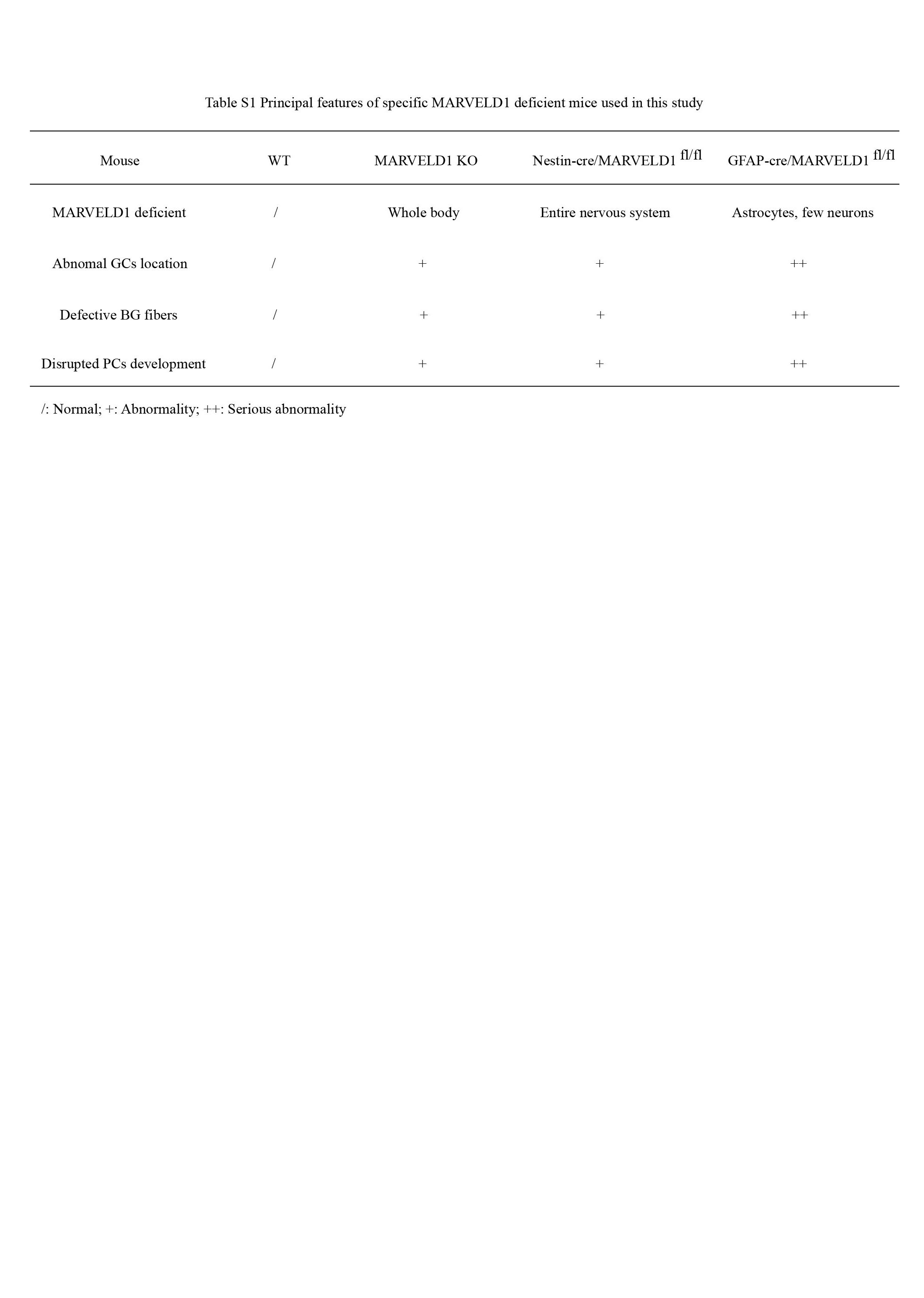

Supplement: Supplementary file 8 — Table S1 [file 41419_2018_1027_MOESM8_ESM.jpg]

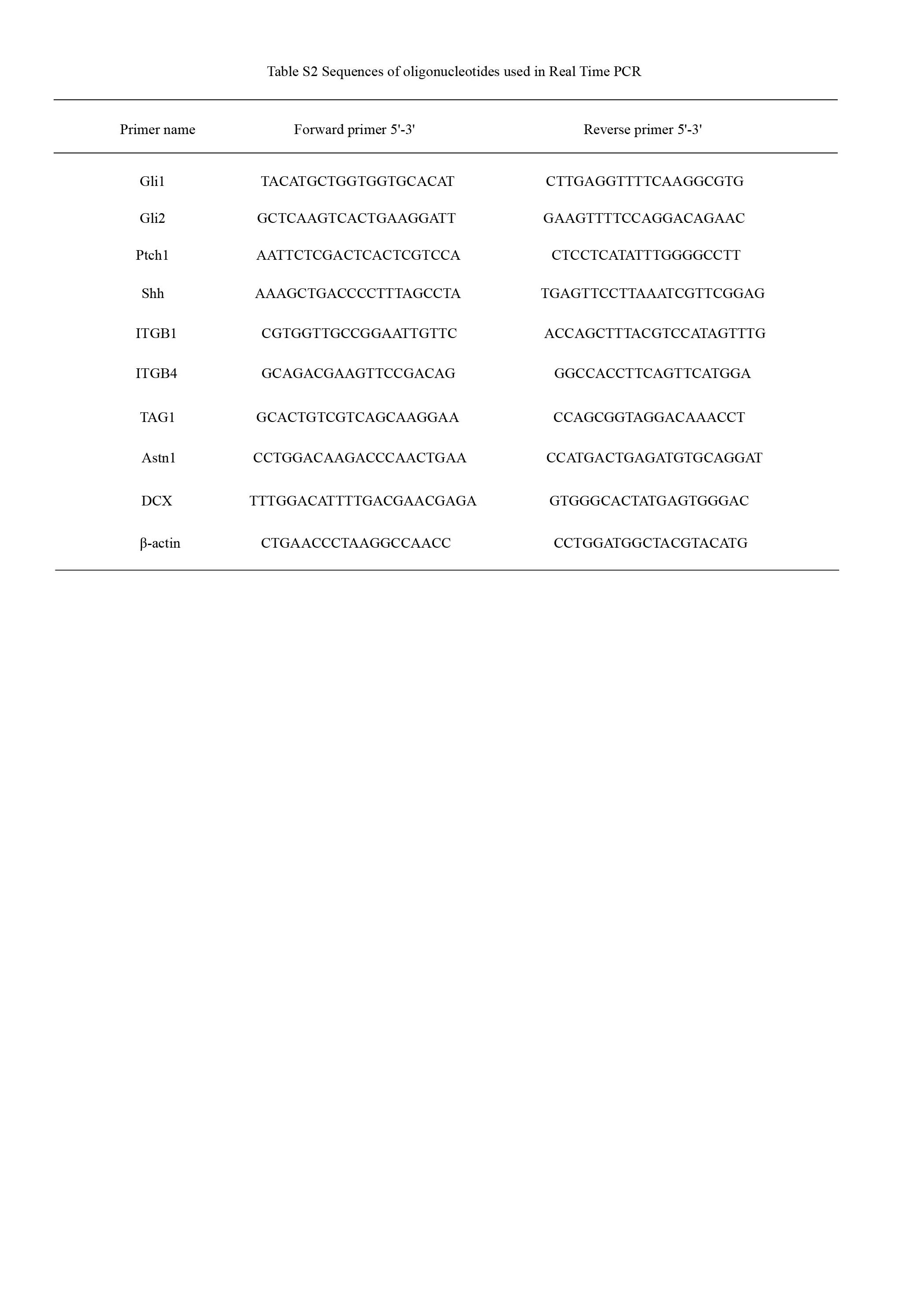

Supplement: Supplementary file 9 — Table S2 [file 41419_2018_1027_MOESM9_ESM.jpg]
